# Sublethal DNA damage switches off B cell effector programs in an RA-FLS-PBMC co-culture

**DOI:** 10.1038/s41420-026-03021-1

**Published:** 2026-03-21

**Authors:** Denada Bruci, Torsten Lowin, Gerhard Fritz, Georg Pongratz

**Affiliations:** 1 Department of Rheumatology and Hiller Research Center, University Hospital Düsseldorf, Heinrich-Heine University Düsseldorf, Düsseldorf, Germany; 2 Institute of Toxicology, Medical Faculty and University Hospital Düsseldorf, Heinrich-Heine University Düsseldorf, Düsseldorf, Germany; 3 Department of Rheumatology, Barmherzige Brüder Hospital, Regensburg, Germany

**Keywords:** Cell death and immune response, Toxicology, Rheumatoid arthritis

## Abstract

Rheumatoid arthritis (RA) features lymphocyte-driven inflammation, in which B cells, alongside T cells, play key effector roles (e.g., autoantibody production, antigen presentation, cytokine or chemokine production). Within activated B cells, during normal diversification, activation-induced cytidine deaminase (AID) introduces targeted DNA lesions in immunoglobulin loci (class-switch recombination/somatic hypermutation), creating a potential vulnerability to sublethal genotoxic stress. T cells also contribute to RA pathogenesis through cytokine production and cell-mediated responses, and are exposed to similar genotoxic stressors in the inflamed joint environment. Given this, we asked whether a single dose/concentration of sublethal genomic damage can modulate lymphocyte effector function without overt cytotoxicity. Peripheral blood mononuclear cells from healthy donors were co-cultured with RA fibroblast-like synoviocytes and exposed once to an IC₂₀ or IC₅₀ dose/concentration of γ-irradiation (γ-IR), hydrogen peroxide (H₂O₂), or the oxazaphosphorine metabolite 4-hydroperoxyifosfamide (4-OOH IFA). Viability, γ-H2AX kinetics, cell cycle status, cytokine and immunoglobulin secretion, and a 28-gene damage response/differentiation panel were quantified at either 24 hours or 5 days post-treatment. Together, the data indicate that a single, carefully titrated low-concentration genotoxic hit might selectively suppress lymphocyte effector programs, with B cells being more durably affected than T cells. At 2 Gy, overall cell viability remained above 80%, whereas IL-10 expression declined by approximately 70%, indicating functional silencing in the absence of substantial cytotoxicity. Conceptually, targeting this vulnerability might help to dampen B cell activity in RA while largely preserving overall immune viability and T cell competence.

## Introduction

Rheumatoid arthritis (RA) is a systemic autoimmune disease that afflicts ~0.5–1% of the world’s population and ranks among the leading causes of disability [1]. Pathologically, it is characterized by synovial hyperplasia, dense leukocyte infiltration, and progressive destruction of cartilage and bone [2]. Although T cell help is indispensable for disease induction, longitudinal histology, serum biomarker studies, and treatment responses now suggest that B cells are pivotal amplifiers of chronic inflammation [3, 4]. Mature B cells accumulate in ectopic germinal centre-like clusters within RA synovium, secrete rheumatoid factor and anti-citrullinated protein antibodies, present antigen to T cells, and elaborate cytokines such as IL-6 and TNF-α [5, 6]. Depletion with the anti-CD20 antibody rituximab validates their clinical relevance; yet, one-third of patients remain refractory, and the risk of infection remains a concern [7, 8]. Consequently, strategies that functionally silence rather than eliminate pathogenic B cells have become an attractive research avenue.

Activated B cells are genetically unique because they engineer their genome; activation-induced cytidine deaminase (AID) deaminates cytosine during somatic hypermutation and class switch recombination, creating programmed double-strand breaks (DSBs) [9, 10]. This inherent genotoxic burden sensitizes these B cells to DNA damage, as assessed by the Ser139-phosphorylated form of the histone variant H2AX (γ-H2AX), a marker of DNA damage [11]. Indeed, B-cell-rich ectopic lymphoid structures are common in RA synovium and are associated with more severe, erosive disease, and phosphorylated γ-H2AX has emerged as a sensitive marker of DNA damage and disease activity in systemic autoimmunity [11, 12]. Moreover, the inflamed joint milieu is a rich source of reactive oxygen species (ROS) and reactive nitrogen intermediates and is often exposed to low-dose ionizing radiation from diagnostic imaging, all of which are potential amplifiers of DNA damage [13–15]. However, whether sublethal lesions alone can recalibrate B cell effector programs without wholescale cytotoxicity remains unresolved.

To explore that question, we employed a human co-culture model of fibroblast-like synoviocytes (FLS) from RA patients and peripheral blood mononuclear cells (PBMCs) from healthy donors. FLS provide contact-dependent and soluble factors that mimic the RA synovial microenvironment [16, 17]. We delivered a single, well-defined dose/concentration (relative IC₂₀ or IC₅₀) of three mechanistically distinct genotoxic agents that are clinically or pathophysiologically relevant to RA: γ-irradiation (γ-IR), used in radiosynoviorthesis and encountered in diagnostic imaging, induces clustered DSBs repaired mainly by Ku-dependent non-homologous end joining (NHEJ) [18]. Secondly, we used hydrogen peroxide (H₂O₂), a diffusible ROS generated not only by activated neutrophils and macrophages in RA joints, but also endogenously by many cell types in inflammatory microenvironments [19]. It causes base oxidation, single-strand breaks, and secondary DSBs among other types of damage, requiring mixed base excision repair (BER), NHEJ, and homologous recombination (HR) repair [20]. Lastly, we used 4-hydroperoxyifosfamide (4-OOH IFA), the active metabolite of the alkylator ifosfamide, structurally related to cyclophosphamide, which may be a necessary therapeutic approach in RA patients with interstitial lung disease or other B cell dependent autoimmune disease, like systemic lupus erythematosus, forms among other types of DNA damage, interstrand cross-links that demand time-consuming HR [21, 22]. Functional, molecular, and viability endpoints were analyzed to determine how genotoxic stress modulates B cell and T cell effector programs within an RA-relevant microenvironment. These insights enabled us to determine the extent to which sublethal genotoxic stress may attenuate B and T cell effector programs while preserving overall viability.

## Results

### Differential short and sustained loss of viability in RA-FLS/healthy PBMC co-cultures after a single γ-IR, 4-OOH IFA, or H₂O₂ challenge

To characterize exposure response and temporal effects, we measured viability at 24 h and day 8 in RA-FLS/healthy PBMC co-cultures following a single graded exposure to γ-IR (Gy), 4-OOH IFA, or H₂O₂. Our methodology used a differential exposure scheme: PBMCs alone were exposed to γ-IR before co-culture, then added to untreated RA-FLS, while 4-OOH IFA or H₂O₂ was applied directly to the co-cultures. Viability measurements refer to the total live-cell population (Annexin V^−^/PI^−^) in the co-culture, including all cell types, PBMCs, and RA-FLS. RA-FLS monocultures contain only RA-FLS cells, allowing specific assessment of their viability independent of PBMCs.

**Fig. 1:**
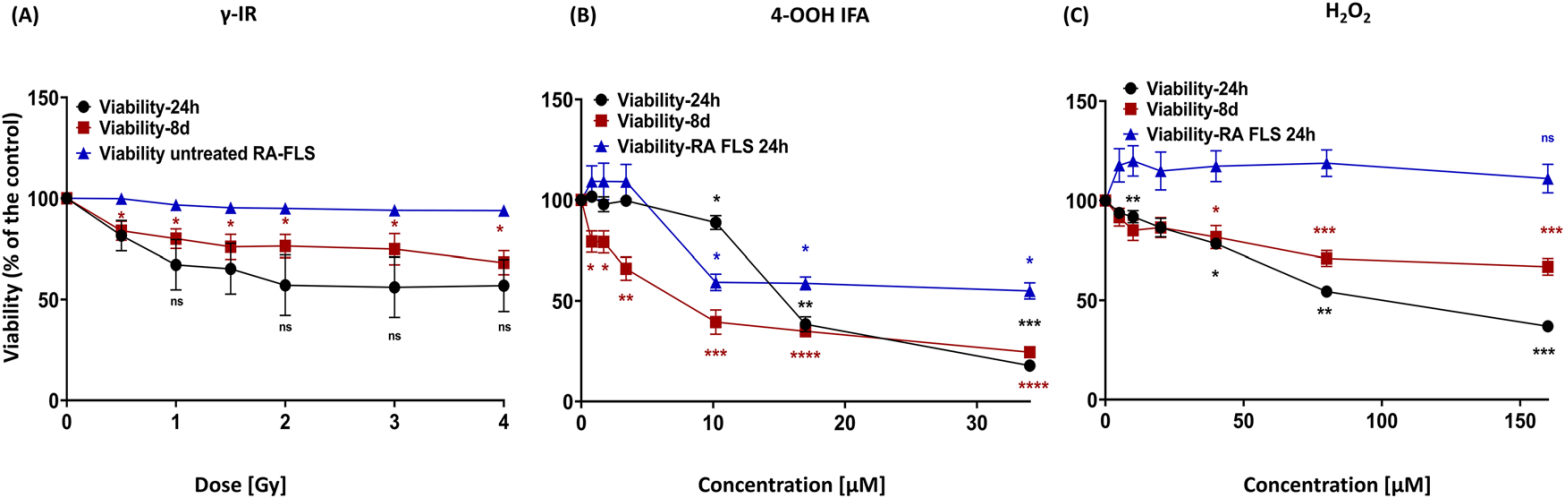
Single dose/concentration genotoxic stress produces distinct short and long-term viability phenotypes in PBMC-RA-FLS co-culture. Dose/Concentration-response of viable cells (Annexin V-/PI-) of whole PBMC-RA-FLS co-culture population at 24 h (black) and day 8 (red) after a single exposure to (**A**) γ-irradiation (0–4 Gy), **B** 4-OOH IFA (0–34 µM), or **C** H₂O₂ (0-160 µM). For (**A**–**C**), RA-FLS-monocultures controls, untreated (**A**) and treated (**B**, **C**), are shown (blue). Dots are mean ± SEM; each donor contributed one measurement per dose/concentration. Values are expressed as % of the untreated control. Four-parameter logistic fits were used to define IC₂₀ and IC₅₀ dose/concentration values. At 24 h, approximate IC₂₀/IC₅₀ values were 0.1/0.4 Gy for γ-IR, 3.5/14 µM for 4-OOH IFA, and 6/23 µM for H₂O₂ (Supplementary Table S2). Statistics: repeated-measures one-way ANOVA with Dunnett’s post hoc vs untreated control. *p ≤ 0.05, **p ≤ 0.01, ***p ≤ 0.001, ****p ≤ 0.0001; ns not significant. PBMC-RA-FLS: *N* = 9, *n* = 1; RA-FLS monoculture, *N* = 3, *n* = 1.

γ-IR induced a monotonic decline in viability of the PBMC-RA-FLS co-culture up to 2 Gy at 24 h, with an estimated IC₂₀ of 0.1 Gy and IC₅₀ of 0.4 Gy; by day 8, the IC₅₀ increased to approximately 0.9 Gy, while the dose response did not change beyond 2 Gy (Fig. 1A and Supplementary Table S2). Despite inter-donor variability limiting post hoc significance, overall ANOVA confirmed a treatment effect. By day 8, both variance and viability further declined (IC₅₀ ≈ 0.9 Gy), but the dose-response did not change beyond 2 Gy, suggesting stable cytotoxicity among surviving co-culture cells. RA-FLS monocultures remained fully viable as they were not irradiated.

4-OOH IFA exposure resulted in the most pronounced loss of viability in the whole co-culture. Statistically significant reductions began at 10 µM (>IC₂₀ of 3.5 µM; IC₅₀ 14 µM) at 24 h, and were amplified by day 8 with an IC₅₀ ≈ 7 µM (Fig. 1B and Supplementary Table S2). High concentrations also impaired RA-FLS monoculture viability (RA-FLS IC₅₀ = 7.7 µM), though RA-FLS were generally more resistant than the PBMC-RA-FLS co-culture at these concentrations. H₂O₂ yielded intermediate effects: a concentration-dependent viability loss of the co-culture was observed from 10 µM upward at 24 h, with an IC₂₀ of 6 µM and an IC₅₀ of 23 µM, but viability partially recovered by day 8 (IC₅₀ of 60 µM). RA-FLS monocultures were unaffected by H₂O₂ (Fig. 1C and Supplementary Table S2). Overall, γ-IR and H₂O₂ caused moderate, delayed cytotoxicity in PBMC-RA-FLS co-cultures, whereas 4-OOH IFA induced rapid and sustained cell loss of the whole co-culture, including partial toxicity to RA-FLS monocultures at high concentrations. These distinct profiles supported the selection of relative IC₂₀ and IC₅₀ doses/concentrations for functional and mechanistic assays. Day 8 was prioritized for effector readouts to capture PBMC-RA-FLS crosstalk and stable mediator secretion, while minimizing the influence of transient early effects.

### Single hit genotoxic stress uncouples lymphokine and immunoglobulin production from cell survival

To evaluate functional consequences independent of early time points, we measured day 8 dose/concentration response production of IL-10, IFN-γ, IL-2, sCD25/IL-2Rα, APRIL, and IgG/IgA/IgM/IgE across γ-IR, 4-OOH IFA, and H₂O₂ treatment of the PBMC-RA-FLS co-cultures. We then used Spearman correlation analyses to relate these effector outputs to corresponding viability measurements, clarifying the relationship between cell survival and functional changes.

**Fig. 2:**
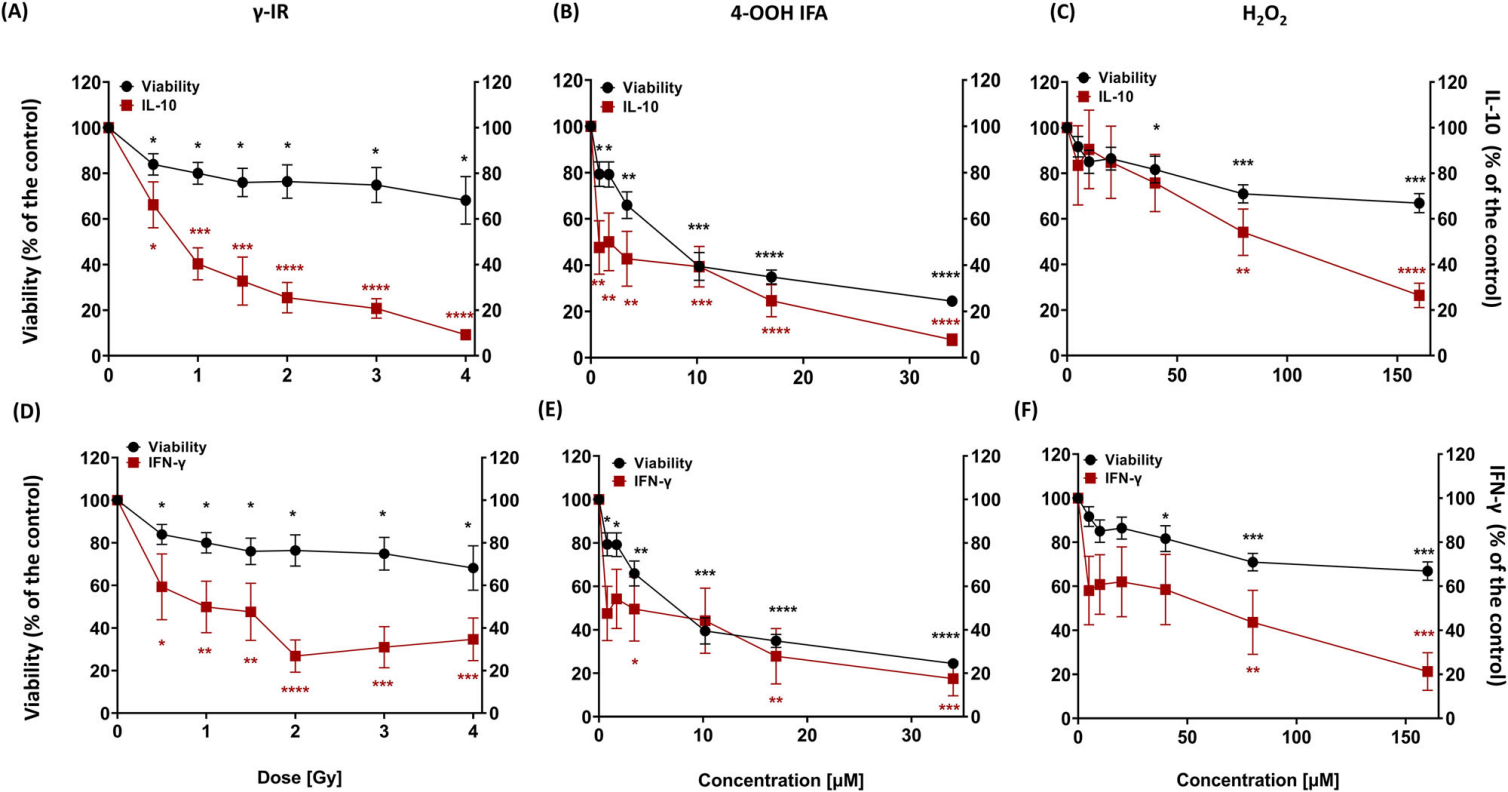
Sublethal DNA damage suppresses IL-10 and IFN-γ secretion more strongly than it reduces cell survival. Day 8 (7 days post-treatment) supernatant dose/concentration responses for IL-10 (**A**–**C**) and IFN-γ (**D**–**F**) in PBMC-RA-FLS co-cultures (red), plotted against the matched day 8 viability (black) of the PBMC-RA-FLS co-cultures from Fig. 1 for γ-IR (**A**, **D**), 4-OOH IFA (**B**, **E**), and H₂O₂ (**C**, **F**). Values are expressed as % of CpG-stimulated untreated control. The viability curve (black line, secondary y-axis) is identical across all panels and represents day 8 viability from the same wells used for the corresponding supernatant measurements. Spearman correlations with viability are summarized in Supplementary Table S4. Statistics: repeated-measures one-way ANOVA with Dunnett’s post hoc vs untreated control. *p ≤ 0.05, **p ≤ 0.01, ***p ≤ 0.001, ****p ≤ 0.0001; ns not significant. PBMC-RA-FLS: *N* = 3, *n* = 3.

**Fig. 3:**
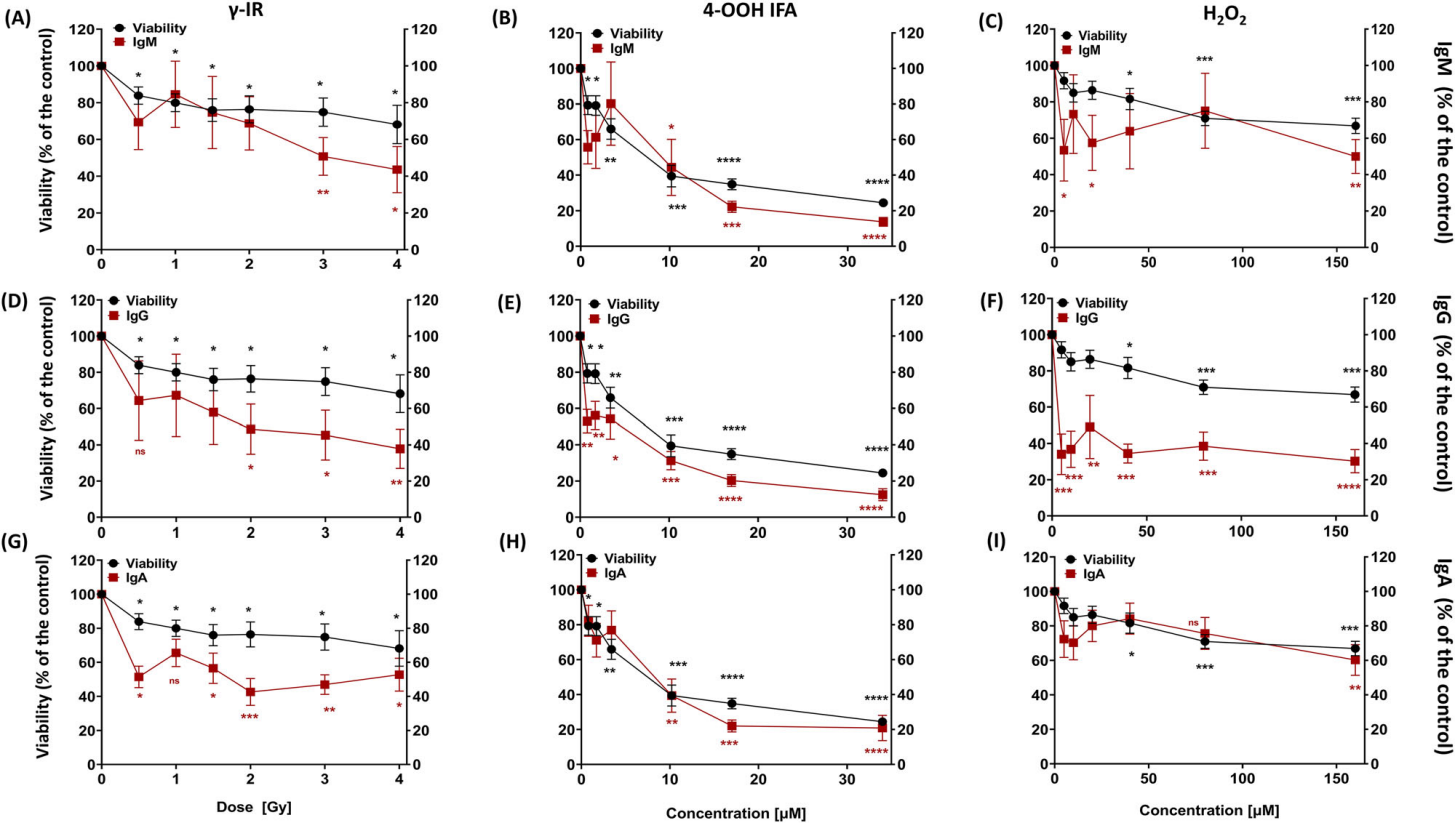
Impact of sublethal genotoxic stress on antibody secretion and cell viability. Day 8 (7 days post-treatment) supernatant dose/concentrations responses for IgM (**A**–**C**), IgG (**D**–**F**), and IgA (**G**–**I**) in PBMC-RA-FLS co-cultures (red), plotted against the matched day 8 viability (black) of the PBMC-RA-FLS co-cultures from Fig. 1 for γ-IR (**A**, **D**, **G**), 4-OOH IFA (**B**, **E**, **H**), and H₂O₂ (**C**, **F**, **I**). Values are expressed as % of CpG-stimulated untreated control. The viability curve (black line, secondary *y*-axis) is identical across all panels and represents day 8 viability from the same wells used for the corresponding supernatant measurements. Spearman correlations with viability are summarized in Supplementary Table S4. Statistics: repeated-measures one-way ANOVA with Dunnett’s post hoc vs untreated control. *p ≤ 0.05, **p ≤ 0.01, ***p ≤ 0.001, ****p ≤ 0.0001; ns not significant. PBMC-RA-FLS: *N* = 3, *n* = 3.

Following γ-IR exposure, IL-10 and IFN-γ secretion were substantially reduced at doses that preserved cell viability, with both cytokines suppressed by approximately 70% at 2 Gy, where 8-day viability remained ≥80% (Fig. 2A, D). IgG and IgA levels were diminished to ∼40–50%, while APRIL, sCD25, and IgM decreased to ∼40–60% of CpG-stimulated controls at 2 Gy (Fig. 3A, D, G; Supplementary Fig. S5A, G). IL-2 was reduced by ∼60% at 1.5 Gy, recovering at higher doses (Supplementary Fig. S5D). Spearman correlation analysis revealed positive associations between cell viability and IL-10, IgG, IgM, and sCD25. In contrast, IFN-γ, IgA, IL-2, and APRIL secretion did not correlate with viability, indicating partial decoupling of specific mediators from cell survival (Supplementary Table S4).

Treatment with 4-OOH IFA elicited an abrupt shutdown of cytokine production, with IL-10 and IFN-γ reduced by over 50% at 5 µM, despite ∼70% viable cells (Fig. 2B, E). At 10 µM, IgG and IgA concentrations were reduced to approximately 40% of control levels, accompanied by ∼50% viability (Fig. 3E, H). Reductions in APRIL and IgM approached 40–60% of control, sCD25 declined to about 30%, and IL-2 to approximately 20% of control (Fig. 3B and Supplementary Fig. S5B, E, H). All immune effectors determined under 4-OOH IFA treatment were positively correlated with cell viability (Supplementary Table S4).

H₂O₂ induced a gradual suppression of function, with cell viability maintained above 90% up to 20 µM. IL-10 decreased by about 30% at 40 µM and transiently converged with the viability curve at 20 µM before separating at higher doses (Fig. 2C). IFN-γ remained consistently below the viability trajectory for the entire concentration range (Fig. 2F). IgG was significantly inhibited at all tested concentrations without concurrent viability loss at lower doses, and parallel declines in both antibody secretion and viability emerged only at concentrations ≥40 µM, while IgA showed only modest, non-significant changes (Fig. 3F, I). In contrast, IgM and sCD25 each dropped to 40–50% of control at 5 µM, APRIL remained approximately 60% of control at 40 µM, and IL-2 declined to 20% of control at the lowest concentration tested (Fig. 3C and Supplementary Fig. S5C, F, I). Correlations between cell viability and IgG, IgA, and IgM weakened under H₂O₂, whereas sCD25, IL-10, IFN-γ, and APRIL retained a positive association; IL-2 secretion was uncoupled from viability (Supplementary Table S4). No IgE production was observed in all three treatments or the control at day 8, while the IgE ELISA positive control yielded a robust signal, confirming assay performance.

In summary, sublethal γ-IR, 4-OOH IFA, or H₂O₂ exposures consistently suppressed B cell-derived immunoglobulin output and accessory factor release, most notably the release of IL-2, a cytokine mainly secreted by T cells that promotes their proliferation and survival. This pattern is consistent with DNA damage checkpoint that may silence immune effector programs at non-cytotoxic concentrations, prompting mechanistic investigation of γ-H2AX accrual in distinct lymphocyte subsets.

### Kinetics of γ-H2AX accumulation reveal lineage-specific DNA damage responses at sublethal and half-lethal doses/concentrations

**Fig. 4:**
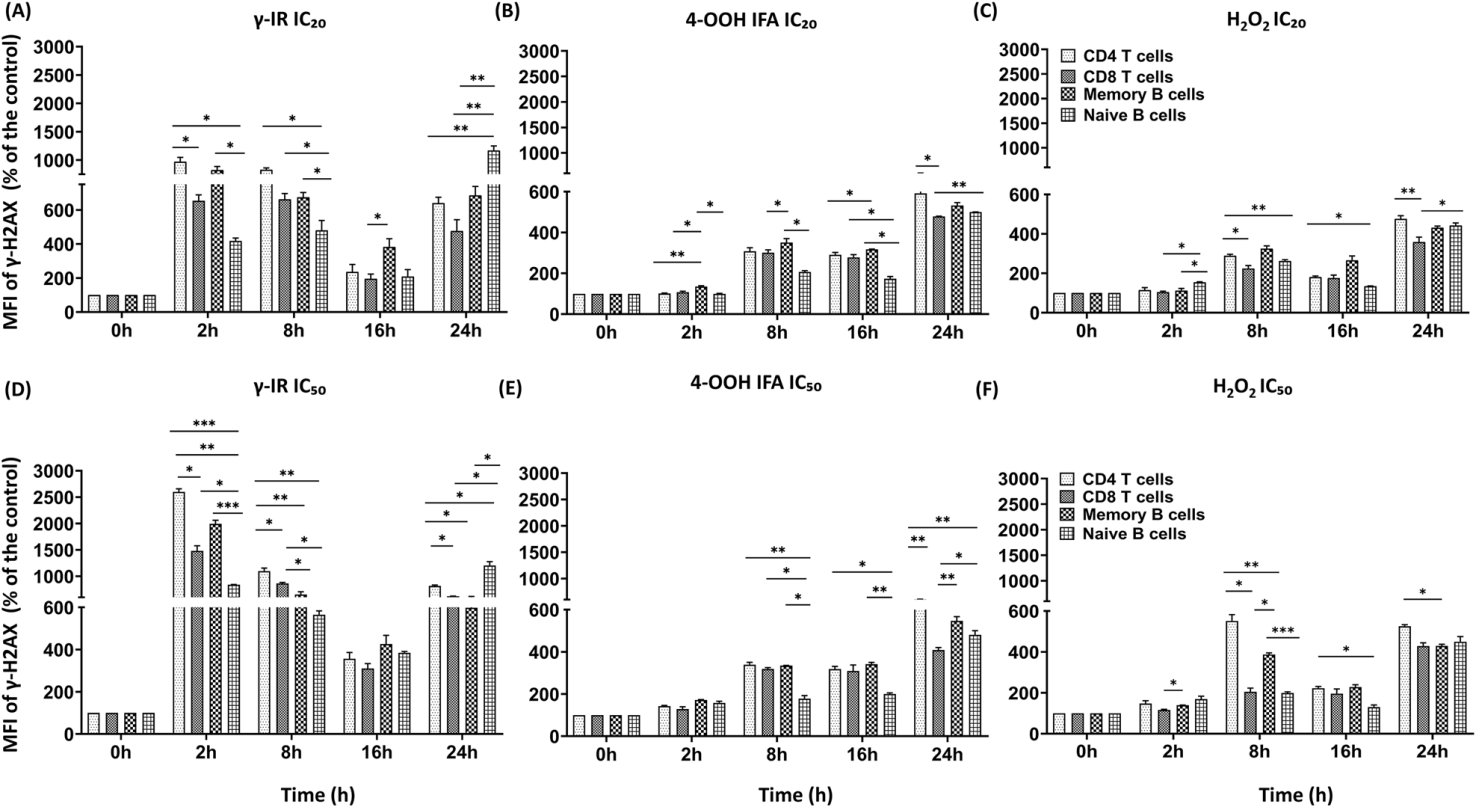
γ-H2AX kinetics reveal lineage-specific DNA damage burdens at IC₂₀ versus IC₅₀. IC₂₀ (**A**–**C**) and IC₅₀ (**D**–**F**) time courses (2, 8, 16, 24 h) of γ-H2AX median fluorescence intensity (MFI) in gated CD4⁺ T, CD8⁺ T, memory B (CD19⁺CD27⁺), and naïve B (CD19⁺CD27^−^) cells within PBMC-RA-FLS co-cultures after γ-IR (**A**, **D**), 4-OOH IFA (**B**, **E**), or H₂O₂ (**C**, **F**). MFI is normalized to the time-matched untreated control. IC₂₀/IC₅₀ doses/concentrations correspond to those derived in Fig. 1 (Supplementary Table S2). Gating strategy: Supplementary Fig. S3; Statistics: repeated-measures one-way ANOVA with Dunnett’s post hoc vs time-matched control at each timepoint. *p ≤ 0.05, **p ≤ 0.01, ***p ≤ 0.001, ****p ≤ 0.0001; ns not significant. Adjusted *p*-values for comparisons to the 0-h baseline within each lineage are reported in Supplementary Table S5. PBMC-RA-FLS: *N* = 4, *n* = 1.

After establishing relative IC₂₀/IC₅₀ concentrations from Annexin V/PI assays that durably suppressed cytokine/immunoglobulin secretion, we next examined DNA damage responses (γ-H2AX accumulation) in lymphocyte subsets. For this, PBMCs were pre-treated with γ-IR (PBMC-alone), while the full PBMC-RA-FLS co-cultures received 4-OOH IFA or H₂O₂ at these doses. At [IC₂₀] (sublethal), γ-IR produced a rapid increase in γ-H2AX across all investigated cell populations, peaking at 2 h and remaining elevated above control at 24 h, indicating incomplete repair (Fig. 4A, Supplementary Fig. S6A and Supplementary Table S5). CD4⁺, CD8⁺ T cells, and memory B cells reached ∼5–7-fold above baseline, while naïve B cells increased to 10-fold at 24 h, as determined by statistical comparison to the 0-h value within each lineage (Fig. 4A and Supplementary Table S5). 4-OOH IFA produced a gradual increase in γ-H2AX, reaching up to 6-fold by 24 h (Fig. 4B, Supplementary Fig. S6B and Supplementary Table S5), and H₂O₂ elicited an early increase at 8 h, a transient dip at 16 h, and a second rise by 24 h (Fig. 4C, Supplementary Fig. S6C and Supplementary Table S5). At [IC₅₀], similar cell-specific patterns were observed but with higher amplitudes. γ-IR triggered 2-h γ-H2AX peaks of 25-fold in CD4⁺, 15-fold in CD8⁺, 20-fold in memory B cells, and 9–10-fold in naïve B cells, decreasing at 16 h yet remaining clearly above baseline in all lineages at 24 h, with the highest residual signal in naïve B cells (Fig. 4D, Supplementary Fig. S6D and Supplementary Table S5). 4-OOH IFA reached comparable levels at 24 h (Fig. 4E, Supplementary Fig. S6E and Supplementary Table S5). H₂O₂ showed biphasic kinetics, with γ-H2AX peaking at 4–6-fold at 8 h and declining to 2–3-fold by 16 h (Fig. 4F, Supplementary Fig. S6F and Supplementary Table S5). Adjusted *p*-values for all cell types/time points are reported in Supplementary Table S5. Basal γ-H2AX MFI levels were higher in naïve B cells compared to memory B cells, CD4⁺ and CD8⁺ T cells (Supplementary Fig. S7), with significant fluctuations over time, even without treatment. All treated sample data were normalized to time-matched controls to enable accurate intra-lineage comparisons. Comparisons of γ-H2AX MFI across treatments at both [IC₂₀] and [IC₅₀] (Supplementary Fig. S8) confirmed the strongest early signals with γ-IR, especially at [IC₅₀]. These analyses indicate that DNA damage signaling might be shaped by both lesion-specific chemistry and cell-intrinsic factors, influencing the dynamics and magnitude of repair responses.

### DNA damage checkpoints results into lineage-specific cell cycle blockade in B cells

Distinct γ-H2AX signaling among lymphoid lineages suggests differential checkpoint engagement with functional consequences. To assess whether these lineage-specific signals affect cell-cycle progression, we analyzed the distributions of CD4⁺ and CD8⁺ T cells, as well as naïve and memory B cells, within PBMC-RA-FLS co-culture at 24 and 48 h following a single sublethal (IC₂₀) or IC₅₀ challenge with γ-IR, 4-OOH IFA, or H₂O₂. This approach links γ-H2AX burden to cell-cycle inhibition and proliferation arrest.

**Fig. 5:**
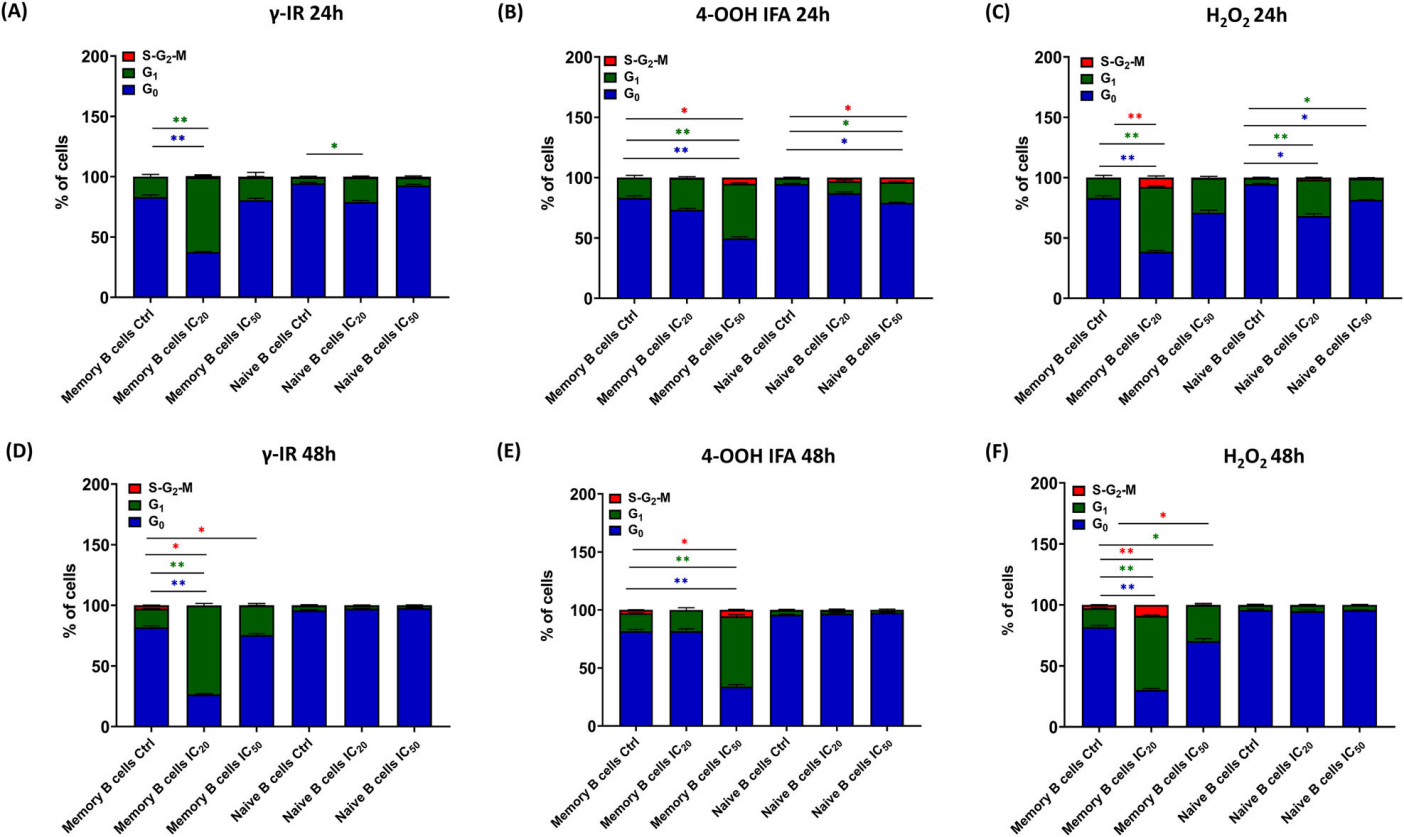
Sublethal genotoxic stress imposes distinct cell cycle checkpoints in memory versus naïve B cells. Ki-67-FITC/Propidium iodide (PI) profiles at 24 h after the single IC₂₀ or IC₅₀ pulse (**A**–**C**) and at 48 h (i.e., 24 h after CpG stimulation; D-F). For each time point, the three stressors are shown side by side: γ-IR (**A**, **D**), 4-OOH IFA (**B**, **E**), and H₂O₂ (**C**, **F**). Within each stressor, the left-hand bars represent memory B cells, and the right-hand bars represent naïve B cells gated within PBMC-RA-FLS co-cultures. Stacked columns display mean ± SEM percentages of cells in G₀ (blue), G₁ (green), and S-G₂-M (red) phases, illustrating checkpoint-mediated accumulation in G₁ with a smaller fraction of cells arrested in S-G₂-M after sublethal genotoxic stress. IC₂₀/IC₅₀ doses/concentrations are as defined in Fig. 1 (Supplementary Table S2). Statistics: repeated-measures one-way ANOVA with Dunnett’s post hoc vs untreated control. *p ≤ 0.05, **p ≤ 0.01, ***p ≤ 0.001, ****p ≤ 0.0001; ns not significant. PBMC-RA-FLS: *N* = 4, *n* = 1.

Ki-67-FITC/PI staining showed that at 24 h post-IC₂₀ γ-IR, memory B cells shifted out of quiescence into G₁, increasing from 18% ± 2% to 64% ± 6%; naïve B cells similarly accumulated in G₁, increasing from 4% ± 1% to 21% ± 4% (Fig. 5A), while IC₅₀ γ-IR produced no significant change. For 4-OOH IFA at [IC₅₀], memory B cells showed G₁ increase from 14% ± 3% to 46% ± 5% and S-G₂-M transition from 0% to 5% ± 1%, with naïve B cells demonstrating modest G₁ and S-G₂-M enrichment (Fig. 5B). H₂O₂ at [IC₂₀] induced the strongest arrest: memory B cells redistributed into G₁ from 15% ± 2% to 55% ± 5% and S-G₂-M from 0% to 10% ± 2%, while naïve B cells increased G₁ to 33% ± 4% at [IC₂₀] and 23% ± 3% at [IC₅₀] (Fig. 5C). Across these conditions, the dominant pattern was a marked accumulation in G₁ with only a small fraction of cells in S-G₂-M.

After 24 h of CpG ODN2006 stimulation (48 h total treatment), naïve B cells in all treatment groups reverted to quiescent status (G₀ > 85%, G₁ < 10%). Memory B cells retained treatment-specific blocks: γ-IR IC₂₀ dose G₁ stayed at 75% ± 6% versus 15% ± 2% in controls, S-G₂-M dropped from 3% ± 1% to 0% (Fig. 5D). 4-OOH IFA [IC₅₀] memory B cells G₁ persisted at 65% ± 5%, S-G₂-M at 7% ± 1% (Fig. 5E). H₂O₂ [IC₂₀] memory B cells G₁ was 62% ± 4%, S-G₂-M at 9% ± 2%, and [IC₅₀] memory B cells G₁ at 34% ± 3% (Fig. 5F). CD4⁺ and CD8⁺ T cells showed only transient G₁ increases (γ-IR IC₂₀) or S-phase spikes (H₂O₂ [IC₅₀]) at 24 h, both resolving by 48 h (Supplementary Fig. S9). These findings indicate that DNA damage-induced checkpoints can establish durable, lineage-specific cell cycle arrest, especially in memory B cells, likely contributing to selective impairment in immune cell proliferation and function.

### Transcriptional responses to DNA damage in PBMC-RA-FLS co-cultures and RA-FLS monocultures

**Fig. 6:**
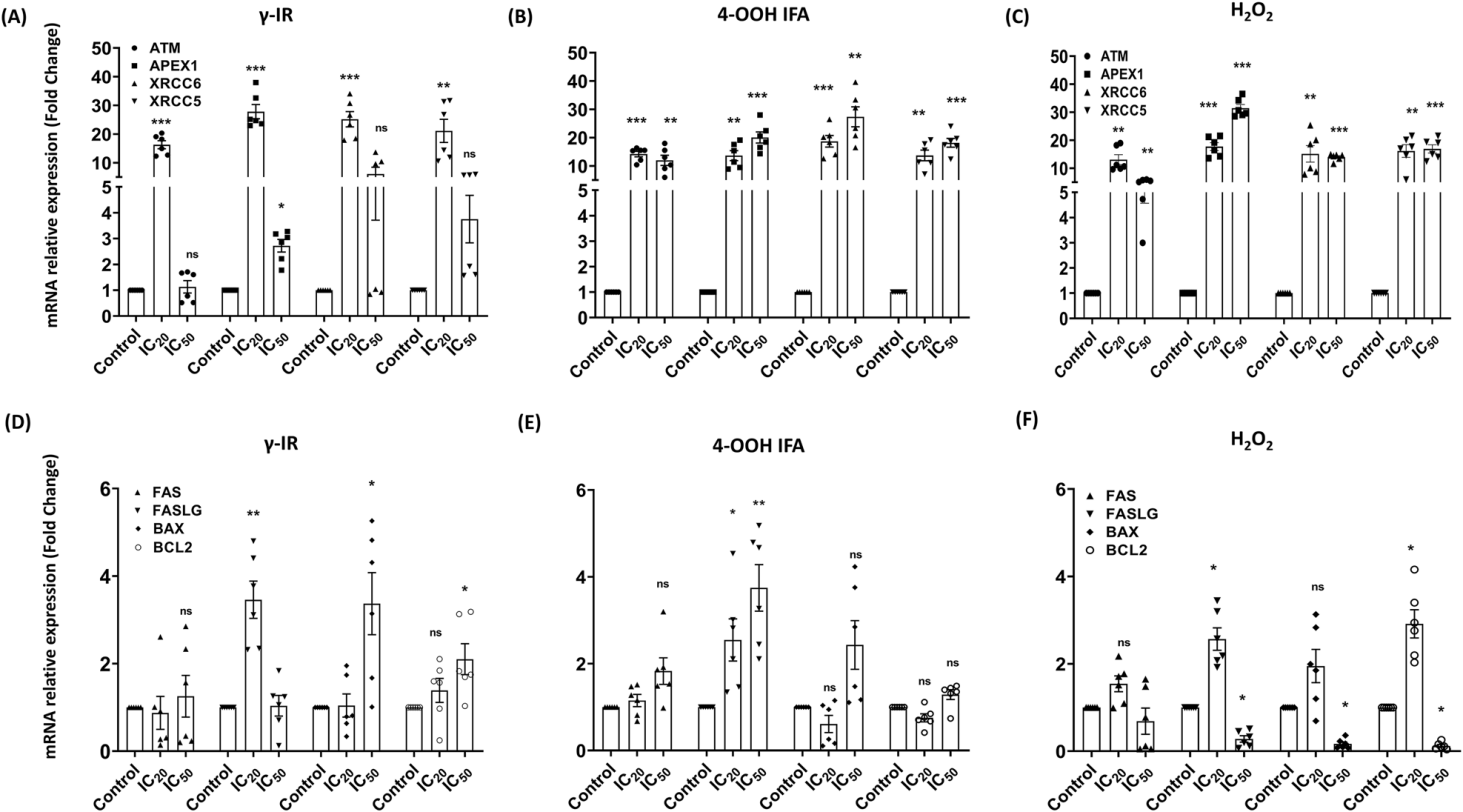
Early transcriptional wiring of the damage response at 24 h. Bar charts show mRNA fold change (ΔΔCt) relative to time-matched untreated control of the whole PBMC-RA-FLS co-culture, measured 24 h after a single IC₂₀ or IC₅₀ dose/concentration of γ-IR (**A**, **D**), 4-OOH IFA (**B**, **E**), or H₂O₂ (**C**, **F**) (mean ± SEM). Upper row (**A**–**C**): DNA damage sensors and repair genes (ATM, APEX1, XRCC6, XRCC5). Lower row (**D**–**F**): apoptosis regulators (FAS/FASLG, BAX, BCL2). IC₂₀/IC₅₀ doses/concentrations follow the viability curves in Fig. 1 (Supplementary Table S2). Statistics: repeated-measures one-way ANOVA with Dunnett’s post hoc vs control. *p ≤ 0.05, **p ≤ 0.01, ***p ≤ 0.001, ****p ≤ 0.0001; ns not significant. PBMC-RA-FLS: *N* = 3, *n* = 2.

**Fig. 7:**
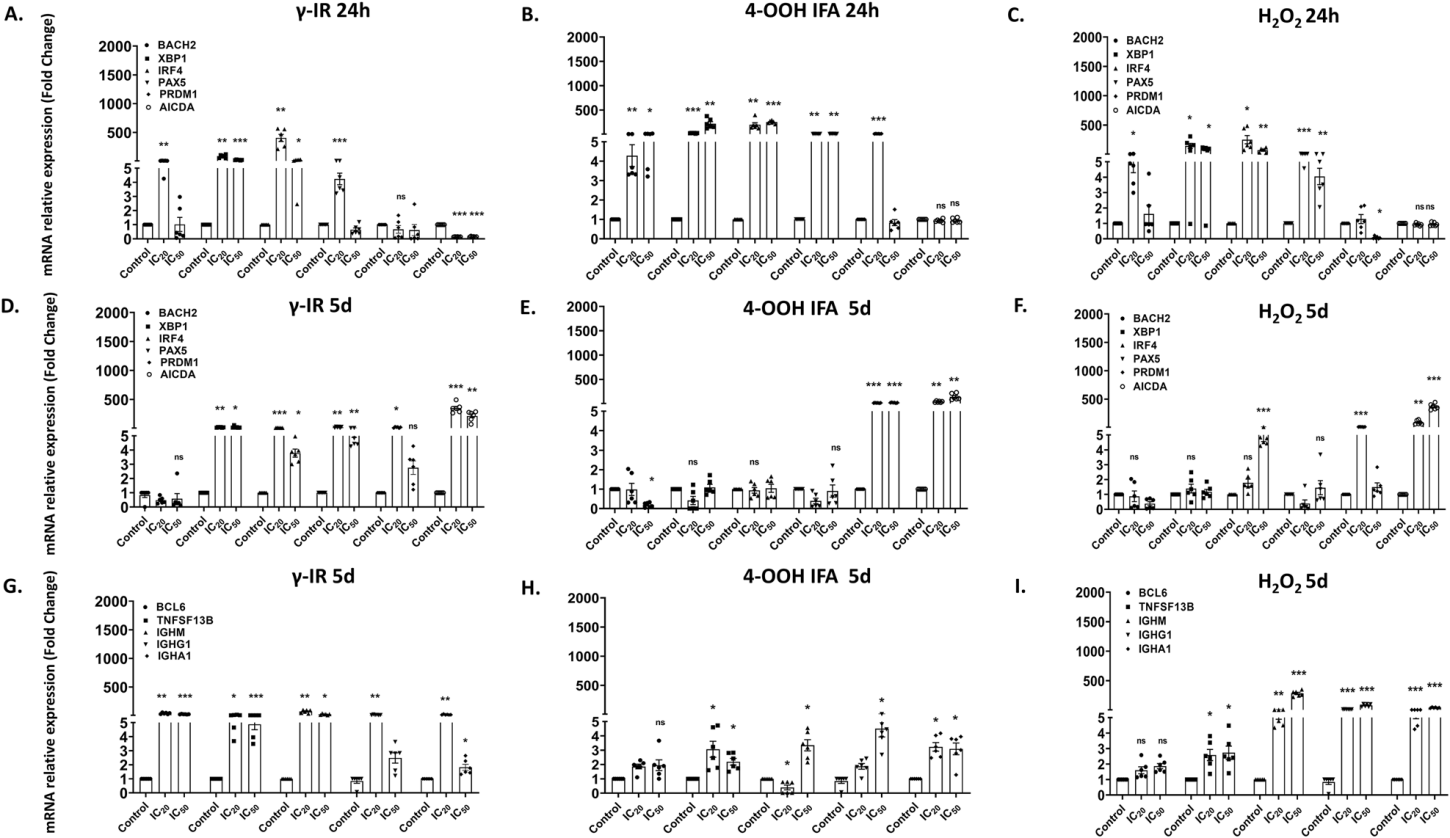
Expression of B cell differentiation, survival genes, and heavy chain transcripts. Bar charts show mRNA fold change (ΔΔCt) relative to time-matched untreated control of the whole PBMC-RA-FLS co-culture, measured 24 h or 5 days (day 6) after a single IC₂₀ or IC₅₀ dose/concentration of γ-IR (**A**, **D**, **G**), 4-OOH IFA (**B**, **E**, **H**), or H₂O₂ (**C**, **F**, **I**) (mean ± SEM). First and second row: BACH2, XBP1, IRF4, PAX5, PRDM1, and AICDA at 24 h (**A**–**C**) and 5 days (**D**–**F**) after IC₂₀ or IC₅₀ doses/concentrations of γ-IR, 4-OOH IFA, or H₂O₂. Third row: BCL6, TNFSF13B, IGHM, IGHG1, and IGHA1 heavy chain transcripts at 5 days post treatment (**G**–**I**) following IC₂₀ or IC₅₀ doses/concentrations of γ-IR, 4-OOH IFA, or H₂O₂. IC₂₀/IC₅₀ doses/concentrations are as defined in Fig. 1 (Supplementary Table S2). Statistics: repeated-measures one-way ANOVA with Dunnett’s post hoc vs control. *p ≤ 0.05, **p ≤ 0.01, ***p ≤ 0.001, ****p ≤ 0.0001; ns not significant. PBMC-RA-FLS: *N* = 3, *n* = 2.

To associate γ-H2AX and cell-cycle checkpoint data with the functional readouts, we profiled 28 transcripts in the whole PBMC-RA-FLS co-cultures and RA-FLS monocultures (Figs. 6, 7 and Supplementary Figs. S10 and S11) by RT-qPCR, collecting mRNA from the co-cultures at 24 h after [IC₂₀] or [IC₅₀] genotoxic challenge, and again on 5 days post-treatment for differentiation markers. At [IC₂₀], all stressors triggered a robust sensor burst in co-culture: ATM, APEX1, XRCC6, and XRCC5 increased by 15–30-fold after γ-IR and 4-OOH IFA, and 15–20-fold after H₂O₂. At [IC₅₀], for γ-IR, the four sensors returned to baseline, whereas 4-OOH IFA and H₂O₂ maintained strong sensor upregulation (10–25-fold) (Fig. 6A–C). RAD51 but not RAD50 was upregulated 4–6-fold at [IC₂₀] and [IC₅₀] for 4-OOH IFA and at IC₂₀ for γ-IR and H₂O₂ (Supplementary Fig. S10). RA-FLS monocultures showed limited transcriptional changes: H₂O₂ selectively increased XRCC5 at 24 h at both IC₂₀ and IC₅₀. 4-OOH IFA elevated APEX1 and ATM at IC₅₀, selectively induced RAD50 at IC₅₀, and increased XRCC5 and RAD51 at both concentrations (Supplementary Fig. S11 A, E).

TP53 transcript was only slightly reduced at 4-OOH IFA [IC₂₀] and H₂O₂ [IC₅₀], while CDKN1A/P21 did not change (Supplementary Fig. S10A–C). BAX increased 3-fold at γ-IR [IC₅₀] but decreased at H₂O₂ [IC₅₀], with no significant change at 4-OOH IFA [IC₅₀]. (Fig. 6D–F). FAS did not change, while FASLG showed specific upregulation with each agent [IC₂₀] in the co-cultures. BCL2 was enhanced 2-fold at γ-IR IC₅₀ and H₂O₂ IC₂₀ dose/concentration but declined at H₂O₂ [IC₅₀] (Fig. 6D–F). BRCA1 and PDCD1 were unchanged, whereas BRCA2 was downregulated at the H₂O₂ [IC₅₀]. BCL6 increased 200–700-fold under all treatments at IC₂₀ and IC₅₀ doses/concentrations. TNFSF13B was upregulated by 10-fold at both doses/concentrations of γ-IR and at 4-OOH IFA by 4-fold at [IC₂₀] and [IC₅₀], with a more limited increase for H₂O₂ at [IC₂₀] (Supplementary Fig. S10D–F). In RA-FLS, H₂O₂ [IC₂₀] significantly induced BCL6, BAX, and TP53, whereas at [IC₅₀] it downregulated BAX and BRCA2. 4-OOH IFA [IC₅₀] elevated BAX, FAS, FASLG, and PDCD1, while at [IC₂₀] it upregulated BCL6, BAX, TP53 and BRCA2 (Supplementary Fig. S11B, C, F, G). These data reveal treatment-specific transcriptomic responses in DNA damage sensors, repair genes, and apoptotic regulators, underlying the observed functional impairments under genotoxic stress.

### Early blockade and late persistence of the B cell differentiation program

To relate the functional loss of Ig secretion to transcriptional checkpoints, we quantified canonical differentiation and plasma cell genes in the whole PBMC-RA-FLS co-culture at 24 h and 5 days post-IC₂₀ or IC₅₀ pulse, focusing on B cell genes due to the clear impact on Ig secretion, and we did not profile T cell differentiation genes in this study.

Twenty-four hours after exposure, all three stressors triggered distinct but overlapping bursts in B cell program genes. γ-IR induced >200-fold rises in XBP1 and IRF4 (both at [IC₂₀] and [IC₅₀]), and BACH2, PAX5 (at [IC₂₀]), with AICDA strongly repressed under both doses (Fig. 7A). 4-OOH IFA drove all five factors to approximately >200-fold at [IC₂₀] and IC₅₀], maintaining high levels of the transcripts, except PRDM1 at [IC₅₀]; AICDA was unchanged. H₂O₂ largely mirrored γ-IR for XBP1, IRF4, and BACH2, with PAX5 steeply elevated also at [IC₅₀]. PRDM1 was repressed at [IC₅₀], and AICDA was unchanged (Fig. 7B, C).

Five days after the single stressor exposure, expression profiles diverged. After γ-IR, XBP1, IRF4, and PAX5 remained ≥200-fold ([IC₂₀]) and ~300-fold ([IC₅₀]); PRDM1 persisted at [IC₂₀], BACH2 returned to baseline, AICDA remained high to >200-fold. With 4-OOH IFA, only PRDM1 and AICDA stayed elevated; others were reduced to almost baseline levels. For H₂O₂, PRDM1 remained high at [IC₂₀], IRF4 showed a modest increase ([IC₅₀]), AICDA surpassed 500-fold at both concentrations, and others stayed near baseline (Fig. 7D–F). In RA-FLS alone (24 h), B cell differentiation genes showed modest, limited changes (Supplementary Fig. S11 D, H).

Gene panels five days after challenge linked germinal center survival signals and class switch recombination to heavy chain transcription (Fig. 7G–I). γ-IR (at IC₂₀/IC₅₀ doses) increased BCL6, TNFSF13B, and µ heavy chain (IgM) transcripts IGHM to ~200–300-fold. IGHG1 and IGHA1 heavy chain transcripts rose sharply at [IC₂₀], with IGHA1 also showing a modest increase at [IC₅₀] (Fig. 7G). For 4-OOH IFA, TNFSF13B transcripts increased at both concentrations, IgM heavy chain transcripts IGHM dropped at [IC₂₀] but rebounded at [IC₅₀], and IGHG1 and IGHA1 heavy chain transcripts increased three to five-fold at [IC₅₀] with IGHA1 already elevated at [IC₂₀] (Fig. 7H). H₂O₂ enhanced transcription of TNFSF13B and all heavy chains transcripts (4–200-fold), with BCL6 unchanged at both concentrations (Fig. 7I). This suggests differentiation blockade and lingering transcriptional shifts, rather than full germinal centre output. These late timepoint data reveal that different genotoxins and γ-IR differentially reactivate B cell effector gene expression, suggesting distinct effects on functional recovery after damage.

## Discussion

This study delineates how a single genotoxic stressor from DNA damage to checkpoint activation impacts immune cell function, especially memory B cells, as shown by γ-H2AX and cell cycle analysis in RA-FLS/healthy PBMC co-cultures. By integrating viability, γ-H2AX kinetics, cell cycle, and transcriptomic analyses, we show that γ-IR, 4-OOH IFA, and H₂O₂ exert distinct effects across immune lineages, especially memory B cells, which seem particularly sensitive to sustained DNA damage signaling and functional shutdown. While classical DNA damage signaling in lymphocytes usually involves ATM and p53 activation, our co-culture system induced ATM but not p53, suggesting established DNA damage models may not fully apply in mixed cultures [23, 24].

### Early versus sustained cytotoxicity: how stressor type sets the tone

Each genotoxic stressor induced a distinct viability trajectory in the RA-FLS/healthy PBMC co-culture. γ-IR produced only modest early death, yet induces a delayed attrition. This delayed death might be due to interphase apoptosis in lymphocytes following irradiation and might reflect the activation of a DNA damage checkpoint [25]. In contrast, 4-OOH IFA caused the steepest and most sustained loss of co-culture cell viability. H₂O₂ had an intermediate effect between these extremes. Early viability dropped, but there was partial recovery by day 8, which might suggest the selection or adaptation of ROS-tolerant cells, consistent with the transcriptional upregulation of antioxidant programs seen in other models [26–28]. The stromal compartment behaved differently. In our design, RA-FLS were not irradiated, so no inference about radioresistance can be drawn. Under 4-OOH IFA, RA-FLS monocultures retained substantially higher viability than PBMC-RA-FLS -rich co-cultures at matched concentrations, but did show impairment at the higher concentrations, indicating relative but not absolute resilience. Using H₂O₂, RA-FLS alone were largely unaffected across the tested concentrations. Therefore, PBMCs seem to be the primary cytotoxic target of all three stressors, whereas RA-FLS are comparatively spared by H₂O₂ and only partly affected by high-concentration 4-OOH IFA, consistent with previous studies demonstrating the resilience of FLSs to oxidative damage [29].

### Functional silencing precedes cell death: a transcriptional checkpoint

Across all three stressors, functional silencing of IL-10, IFN-γ, IgG, and IgA routinely occurred before overt cytotoxicity, suggesting regulation at a transcriptional checkpoint while cells remained viable. This pattern is consistent with an established DNA damage response hierarchy, in which ATM sensor activation can promote chromatin changes and transient repression of differentiation programs, including the AICDA-PRDM1-XBP1 axis, thereby prioritizing repair over specialized immune functions [30–32]. Our findings may support this mechanism, as robust induction of ATM and associated sensors occurred rapidly, with only modest changes in apoptotic genes and cell-cycle effectors [30, 33]. However, positive correlations between immune effector production and viability also indicate a relationship with cell death and thus cannot, on their own, exclude a model in which protein loss results solely from cytotoxicity. Indeed, the inverse correlation between cell survival and effector loss could reflect either checkpoint-regulated suppression or progressive cell death. The most rigorous demonstration of independent checkpoint regulation would require identifying a factor positively associated with cell death or specifically upregulated in surviving cells post-stress, a criterion that DNA repair factors might fulfill if de novo synthesis coincides with increased cell death. Our transcriptomic data show that ATM, APEX1, XRCC6, and XRCC5 are rapidly upregulated after stress, which is compatible with this model [25, 28, 33, 34].

Agent-specific chemistries further shape the depth and breadth of functional silencing. γ-IR predominantly activates ATM signaling and is associated with a relatively moderate but sustained suppression of gene expression, whereas replication-blocking lesions such as 4-OOH-IFA are expected to elicit a strong ATR-CHK1 response with broader effects on S-phase transcriptional programs, and H₂O₂ engages mixed oxidative and base damage as well as redox-responsive pathways that can modulate transcription, as indicated by previous studies [35–37]. Notably, IL-2 demonstrates regulation independent of viability, implicating additional pathways (e.g., NF-κB, AP-1, STATs) beyond ATM-driven checkpoints [38–41]. Antibody isotypes also showed distinct regulation. IgG and IgA were more sensitive to early functional suppression than IgM, consistent with the metabolic demands of class-switch recombination (CSR), dependent on AID and ER/UPR programs tightly monitored by the DNA damage response. Basal IgM production, being less energetically demanding, was relatively preserved, while CSR and high-level antibody output were preferentially attenuated at the protein level, especially at low concentrations/doses of γ-IR and H₂O₂, even though heavy-chain transcripts (IGHG1, IGHA1, and IGHM) often remained detectable or were upregulated. These patterns corroborate prior findings and reinforce a model of stress-dependent transcriptional and post-transcriptional throttling rather than complete shutdown [42–46]. Given that these class-switched isotypes predominantly arise from memory B cells, and our γ-H2AX and cell cycle data substantiate vulnerability in this subset, we infer that the observed isotype suppression under genotoxic stress is likely driven primarily by memory B cell checkpoint engagement. However, as naïve B cells can also be activated and undergo CSR in response to CpG stimulation, we cannot fully exclude their contribution to the observed effects. Quantifying the ratio of reactivated memory to newly activated naïve B cells would be required to substantiate this inference; however, it remains interpretive yet central to our model [47–50].

Overall, robust sensor induction and selective effector suppression at sublethal exposures, in the absence of strong apoptotic signals, are most consistent with early checkpoint regulation as the principal mechanism, rather than cell death per se. At the same time, the positive Spearman correlations between viability and IL-10/IgG/IgM/sCD25, particularly for 4-OOH-IFA, might indicate that emerging death- or senescence-associated programs begin to contribute as concentration increases within the low-concentration window. Thus, functional silencing appears to precede and then gradually converge with regulated cell death pathways, rather than being completely independent of them. This aligns with increasing evidence that DDR pathways can directly downregulate immune effector gene expression via ATM/ATR signaling before apoptosis is initiated [31, 51, 52]. While our study cannot disambiguate single-cell checkpoint effects from population-level coordination, the observed association between repair factor induction and cell death strengthens the case for differential regulation in this co-culture setting, which may resemble key aspects of the inflamed RA joint environment.

### Lesion kinetics and cell cycle checkpoint responses

Our kinetic analyses revealed stressor-specific γ-H2AX and cell cycle checkpoint engagement, with prolonged and synchronous activation particularly in memory B cells (identified by flow cytometry) within the PBMC-RA-FLS co-culture. γ-IR drove a rapid and synchronous γ-H2AX peak across lymphoid lineages, with T cells displaying the highest early activation and swift resolution, whereas naïve B cells exhibited sustained γ-H2AX at late timepoints, suggesting slower repair capacity. In contrast, 4-OOH IFA produced a delayed, monotonic γ-H2AX increase, consistent with interstrand crosslink damage and prolonged checkpoint activation described for similar agents. H₂O₂ triggered a biphasic γ-H2AX response, resulting in mixed and transient cell cycle checkpoint patterns [53]. Notably, 4-OOH IFA was associated with prolonged checkpoint engagement, as reflected by delayed RAD51 induction in the whole co-culture and persistent γ-H2AX in memory B cells, coinciding with selective loss of antibody output and differentiation markers [18, 54]. H₂O₂, by contrast, allowed functional recovery following checkpoint resolution. These findings highlight a nuanced picture: memory B cells are particularly vulnerable to prolonged checkpoint activation and functional suppression under genotoxic conditions, a central finding of our study. Although an initial DNA damage checkpoint likely attenuates antibody secretion, the damage appears to be repaired or tolerated sufficiently, allowing CpG stimulation to activate the secretory program, including class-switch output at later time points [27, 53]. This transient, stress-adapted phenotype aligns with studies showing that efficient repair of oxidative damage might enable cellular recovery and renewed function [27, 53, 55].

Some limitations need to be discussed, and also highlighted clear avenues for future work. Direct measurement of DNA lesion spectra, repair kinetics, and protein-level validation in disease-relevant single-cell assays would further strengthen the mechanistic interpretation, but our current approach, leveraging population-level analyses in pathophysiologically relevant co-cultures, already supports coordinated DNA damage checkpoint engagement and immune suppression in an inflammatory-like niche. The inclusion of PBMC-RA-FLS co-cultures introduces the potential for paracrine effects and more closely reflects in vivo immune-stromal interactions, an essential consideration for RA pathogenesis. Our conclusions are presently confined to healthy PBMCs in an RA-FLS-supported niche; we did not systematically assess PBMC monocultures, autologous RA PBMC-RA-FLS pairs, or co-cultures with non-RA fibroblasts, which may reveal additional cell-intrinsic or stromal context-dependent differences and represent important extensions for future studies. PBMC monocultures were not analyzed in depth because they failed to survive long-term without stromal support, as illustrated in Supplementary Fig. S12, which shows rapid loss of viability and minimal survival and cytokine production at day 8. In addition, we did not perform ATM/ATR inhibitor or genetic perturbation experiments, so DDR dependence is inferred from converging markers (γ-H2AX kinetics, checkpoint phenotypes, DDR transcript induction) rather than demonstrated by direct kinase inhibition, and such targeted mechanistic studies are a logical next step to refine the pathway-mechanistic mode.

**Fig. 8:**
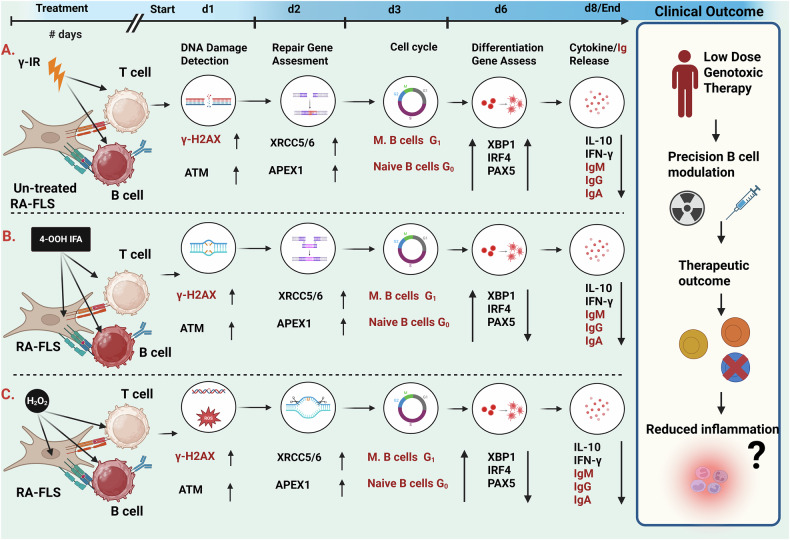
Genotoxic stress responses over time in PBMC-RA-FLS co-culture. **A**–**C** trace the time-ordered cellular responses to **A** γ-irradiation (γ-IR), **B** 4-hydroperoxyifosfamide (4-OOH IFA), and **C** hydrogen peroxide (H₂O₂). In **A** (γ-IR), treatment was confined to the PBMC fraction (T and B cells among other cells), whereas in **B**, **C**, the entire PBMC-RA-FLS co-culture was exposed. Each pathway begins with DNA damage detection (as indicated by γ-H2AX and ATM induction in the PBMC-RA-FLS population), followed by engagement of Repair Programs (e.g., APEX1 and XRCC5/6). By day 3, prolonged and synchronous cell cycle checkpoint activation is evident in memory B cells (identified by flow cytometric differential gating within the PBMC population), resulting in G₁ arrest. Transcriptional programs rebound by day 6 (5 days post-treatment), showing stressor-dependent regulation of differentiation regulators (IRF4, XBP1, PAX5) and immunoglobulin genes within PBMC-RA-FLS co-culture. By day 8, functional silencing is evident, as indicated by reduced cytokine and immunoglobulin secretion (cytokines from the whole co-culture; immunoglobulins from B cells). The red labels correspond to the B cell population within the PBMCs. The inset panel (right) illustrates a proposed clinical application: a precisely titrated, low-dose genotoxic exposure might dampen pathogenic B cell activity while sparing stromal fibroblasts and T cell compartments, potentially opening a therapeutic avenue for rheumatoid arthritis and other B cell-driven conditions, although this application remains hypothetical and was not directly tested in our study. Together, these data outline a testable framework for future studies of genotoxic immunomodulation. This figure was created using BioRender, used with permission.

In summary, our findings suggest that sublethal, type-dependent genotoxic stress can selectively engage DNA damage checkpoints in memory B cells while largely preserving overall cell viability in a physiologically relevant PBMC-RA-FLS co-culture. These key findings are summarized schematically in Fig. 8. By characterizing these coordinated responses, this study contributes to a clearer understanding of how genotoxic stress affects immune function and establishes a foundation for future investigations in disease contexts and more refined cell populations.

## Conclusion

In summary, we describe a coherent sequence linking DNA damage (measured by γ-H2AX kinetics) and associated sensor gene expression to cell cycle checkpoint engagement and subsequent transcriptional alterations, which may help explain how DNA damage selectively recalibrates B cell function in this co-culture model. These findings may provide a rationale for exploring precision, low-concentration genotoxic modulation of target cells in autoimmune diseases, suggesting that a brief exposure to an alkylator or radiotherapeutic agent might silence pathogenic B cells while leaving stromal fibroblasts and most T cells relatively intact. Mapping this cascade offers a conceptual basis for future strategies that might couple genotoxic agents with targeted immune modulation in rheumatoid arthritis and related B-cell-driven disorders.

## Materials and methods

### Cell culture and co-culture setup

FLS were isolated from synovial tissue of patients with RA after informed consent and ethics approval (Study-Nr.: 2022-2189_7) [56]. Mycoplasma negativity was routinely confirmed. Cells were expanded to passages 3–5 and seeded at 1 × 10⁵ cells per well in 48-well plates. Cultures were maintained in RPMI 1640 (Sigma-Aldrich) supplemented with 10% fetal bovine serum (FBS; Gibco), 1% GlutaMAX (Thermo Fisher Scientific), 1% sodium pyruvate (Thermo Fisher Scientific), 1% penicillin-streptomycin (Thermo Fisher Scientific), and 10 mM HEPES (Thermo Fisher Scientific) at 37 °C, 5% CO₂. After 24 h, PBMCs from healthy donors were added. Healthy donor PBMCs were obtained from male donors aged 25–50 years with no history of autoimmune disease or acute infection, and donors provided signed informed consent (Probandeninformation und Einwilligung). No donor samples were excluded from analysis. PBMCs were isolated by Lymphoprep density-gradient centrifugation (Progen), followed by red blood cell lysis, then washed, counted, and added to RA-FLS at a 1:5 ratio (1 RA-FLS:5 PBMCs) in the same medium.

### Genotoxic challenge and stimulation scheme

Co-cultures or only PBMCs were treated with the following reagents: PBMC alone with γ-IR (0–4 Gy) delivered using the Gammacell 1000 Elite radiation machine (Nordion International, and the PBMC-RA-FLS co-cultures with 4-OOH IFA (0–34 µM) obtained from Niomech IIT GmbH, and H₂O₂ (0–160 µM) purchased from Sigma-Aldrich. After 24 h, half of the wells were harvested (acute endpoint). The remaining wells were stimulated with CpG ODN 2006 (5 µg/mL; Invivogen) before further incubation until day 8. The complete treatment scheme is shown in Supplementary Fig. S1. All compounds in this study are listed in Supplementary Table S1. Treatment conditions were randomized across wells of multi-well plates to avoid positional batch effects; no row or column was dedicated to a single condition.

### Cell viability assay

Cell viability (defined as the Annexin V-negative/PI-negative population) was assessed by Annexin V-FITC/PI staining (Miltenyi Biotec) according to the manufacturer’s protocol and analyzed on CytoFLEX LX flow cytometer (Beckman Coulter) at 24 h and 7 days post-treatment (day 8) (Supplementary Fig. S2 for gating). To determine sublethal working doses/concentrations, PBMCs alone received graded γ-IR (0–4 Gy) and were added on top of untreated RA-FLS. Furthermore, PBMC-RA-FLS co-cultures received 4-OOH IFA (0–34 µM), or H₂O₂ (0–160 µM). Four-parameter logistic regression yielded relative IC₅₀ values of the whole co-culture (Supplementary Table S2). From the same fits, IC₂₀/IC₅₀ values at 24 h were approximately 0.1 Gy/0.4 Gy for γ-IR, 3.5 µM/14 µM for 4-OOH IFA, and 6 µM/23 µM for H₂O₂. Unless stated otherwise, IC₂₀ and IC₅₀ refer to relative effect levels, defined as the concentrations producing 20% and 50% of the maximal viability reduction observed across the dose/concentration response range of the PBMC-RA-FLS co-culture, respectively, i.e., sublethal benchmarks. Relative IC₅₀/IC₂₀ values were calculated for the entire PBMC-RA-FLS co-culture population. All compounds and antibodies used in this study are listed in Supplementary Table S1.

### Cytokine and immunoglobulin quantification by ELISA

ELISA kits were purchased from R&D Systems for cytokines and chemokines (e.g., IL-10, IFN-γ, APRIL, sCD25/IL-2 Rα, IL-2) and StemCell Technologies for immunoglobulins (IgM, IgG, IgA, IgE). Assays were performed according to the manufacturer’s protocols. Absorbance was measured at 450 nm using a Tecan Infinite M200 Pro microplate reader (Tecan), and the data were analyzed using Microplate Manager software (Bio-Rad). Cytokine and immunoglobulin values are expressed as a percentage of the CpG-stimulated, but otherwise untreated, control and plotted against the corresponding day 8 viability obtained from the same wells. All used kits are found in Supplementary Table S1.

### Flow cytometric assays

Common preparation: Co-cultured PBMC-RA-FLS cells were detached with Accutase (10 min, 37 °C), washed in FACS buffer (PBS, 3% FBS, 2 mM EDTA), blocked with FcR reagent (1:5, Miltenyi; 15 min, 4 °C), and stained with surface markers (20 min, RT). Lineages were defined using: CD19-eFluor 405 (Invitrogen; 1:20), CD4-PerCP/Vio700 (1:50), CD8-VioGreen (1:50), CD27-VioBright R720 (1:50), and CD3-PE/Vio770 (1:50). Cells were fixed in 4% paraformaldehyde (Thermo Fisher Scientific; 15 min, RT) and permeabilised in 0.1% Triton X-100 (Thermo Fisher Scientific; 10 min, RT), then stained for intracellular targets in PBS with 0.05% Tween-20 and 3% FBS. Data were acquired on a CytoFLEX LX (Beckman Coulter) and analyzed in FlowJo v10.8.1. All compounds and antibodies used in this study are listed in Supplementary Table S1.

#### γ-H2AX assay for DNA Damage

After surface staining, fixation, and permeabilization, cells were incubated with anti-γ-H2AX-Alexa Fluor 488 (BD Biosciences, 1:20) for 1 h at RT. It was followed by a wash step, after which it was resuspended in FACS buffer. Gating strategy is outlined in Supplementary Fig. S3.

#### Ki-67 and PI cell cycle assay

After permeabilization, cells were incubated with Ki-67-FITC (Miltenyi Biotec, 1:50) for 20 min at room temperature in the dark. They were then counterstained with PI (propidium iodide, Miltenyi Biotec, 1:100) for 20 min at room temperature in the dark. Gating strategy is outlined in Supplementary Fig. S4. All compounds and antibodies used in this study are listed in Supplementary Table S1.

### Real-time quantitative PCR (RT-qPCR)

Total RNA was extracted from co-cultured PBMC-RA-FLS cells or RA-FLS only, at 24 h or 5 days post-treatment (day 6), using the NucleoSpin RNA isolation kit (Macherey-Nagel) following the manufacturer’s instructions. Reverse transcription was performed using the High-Capacity cDNA Reverse Transcription Kit (Applied Biosystems). qPCR was performed with SYBR Green Master Mix (Applied Biosystems) to quantify the expression of BCL6, TNFSF13B, AICDA, and other target genes. 18S rRNA was used as the reference gene, and relative gene expression was calculated using the ΔΔCt method. The primer sequences are given in Supplementary Table S3, and comprise in‑house designed and literature‑derived primer pairs (e.g. Ouzin et al. 2024) [57]. RA-FLS are not antibody-producing cells; immunoglobulin and AICDA/GC-factor panels are included to document baseline/indirect transcriptional effects in the stromal compartment.

### Statistical analysis

Data are expressed as mean ± standard error of the mean (SEM). For viability, DNA damage, cell cycle, cytokine/chemokine/immunoglobulin (day 8), and RT-qPCR analyses, we used repeated-measures one-way ANOVA with Dunnett’s post hoc test to account for the paired and matched nature of samples derived from the same donors across multiple conditions. Significance thresholds: *p* ≤ 0.05 (*), *p* ≤ 0.01 (**), *p* ≤ 0.001 (***), *p* ≤ 0.0001 (****), ns: not significant. Spearman correlations assessed monotonic dose/concentration-viability/cytokine relationships (Supplementary Table S4). Variance similarity between groups was confirmed by visual inspection of SEM bars and Prism’s Brown-Forsythe test (*p* > 0.05). Within-donor effects were displayed as SEM, and between-donor variability was controlled by a repeated-measures design. The Shapiro-Wilk test confirmed normality suitable for parametric tests (*p* > 0.05). Analyses were performed in GraphPad Prism 8 using independent donor material (*N* = 3–9 donors). All experiments used independent donor material. The language and grammar of this manuscript were checked using language editing assistance, with all content, data interpretation, and conclusions generated solely by the authors.

## Supplementary information

Supplementary Material

## Data Availability

The data supporting the findings of this study are available from the corresponding author upon reasonable request.
