# Supplementary material for: Sublethal DNA damage switches off B cell effector programs in an RA-FLS-PBMC co-culture
Source: Cell Death Discov. 2026 Mar 21;12:161. doi: 10.1038/s41420-026-03021-1 (PMC13039513; doi:10.1038/s41420-026-03021-1)
Supplement: Supplementary file 1 — Supplementary Material [file 41420_2026_3021_MOESM1_ESM.pdf]

**Supplementary Figures:**

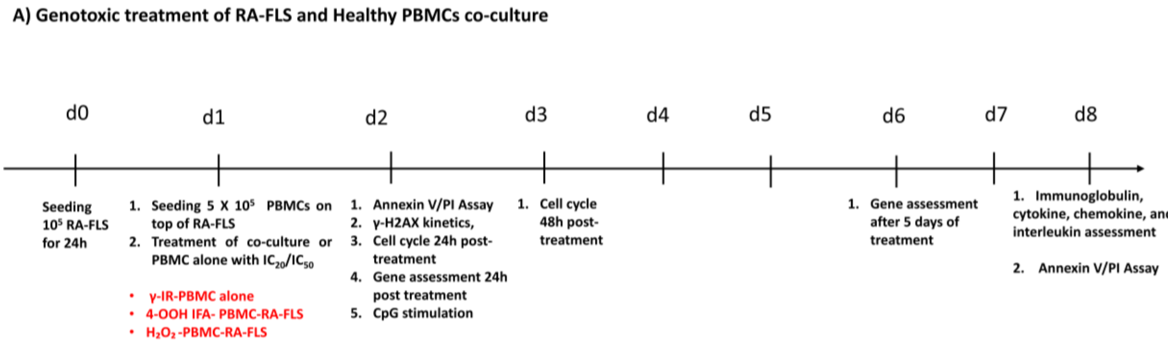


**Supplementary Figure S1. Experimental timeline and sampling scheme.** RA-FLS were seeded 24 h before treatment (day 0). On day 1, freshly isolated healthy-donor PBMCs were added to RA-FLS at a 1:5 (RA-FLS: PBMC) ratio. Genotoxic stress was applied as follows: for the alkylator and oxidant, 4-OOH IFA or $H_2O_2$ (at concentrations spanning the empirically determined [$IC_{20}$] and [$IC_{50}$]) was added directly to the whole co-culture; for the irradiation part, PBMCs were γ-irradiated separately and then immediately transferred onto RA-FLS (RA-FLS were not irradiated). After 24 hours post-treatment, whole PBMC-RA-FLS co-culture cells were harvested for Annexin V/PI assay, the first RT-qPCR panel (damage sensors/repair and early B cell differentiation genes), and for Ki-67-FITC/PI cell-cycle analysis. DNA damage (γ-H2AX, flow cytometry) of different lineages within PBMC-RA-FLS co-culture was profiled between 0 and 24 hours post-treatment (2, 8, 16, 24 hours). Immediately after the 24-hour read-outs, the co-cultures were stimulated with CpG-ODN2006; a second Ki-67-FITC/PI measurement was acquired 24 hours post CpG treatment (i.e., 48 hours from treatment). A delayed RT-qPCR harvest of the whole PBMC-RA-FLS co-culture was performed on day 6 (5 days post treatment) to capture slower transcriptional programmes, and co-culture supernatants were collected on day 8 for ELISA (cytokines and immunoglobulins). At this final time point,

Annexin V/PI was also performed to assess late viability. All measurements were normalised to time-matched untreated controls; additional details (exact concentrations, replicates, and statistics) are provided in Materials and Methods.

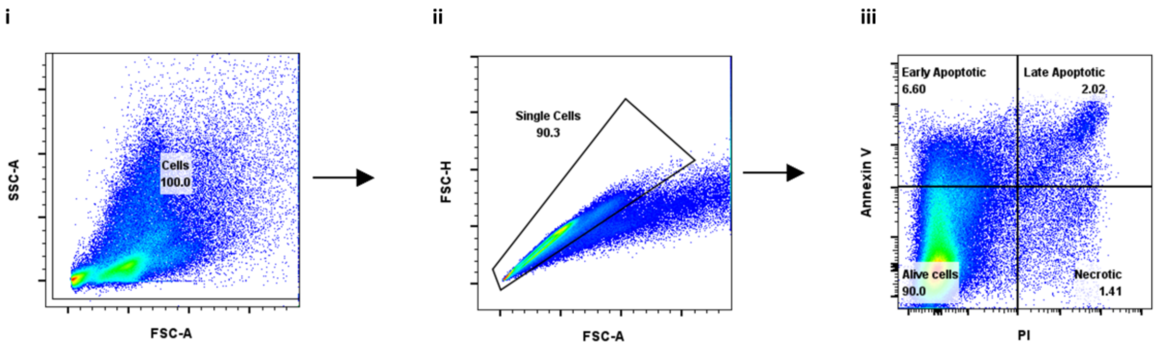


**Supplementary Figure S2: Flow cytometry gating strategy for Annexin V vs PI assay (illustrative, no statistics).** (i) FSCA × SSCA to select the total cell population and exclude debris, (ii) FSCH × FSCA doublet discrimination gate, (iii) Annexin V-FITC/PI gates were used to quantify alive (Annexin V$^-$/PI$^-$), early apoptotic (Annexin V$^+$/PI$^-$), and late apoptotic (Annexin V$^+$/PI$^+$), and necrotic cells (Annexin V$^-$/PI$^+$), in RA-FLS/healthy PBMC co-cultures. The viable population (Annexin V$^-$/PI$^-$) provided the percentage values plotted in central Figure 1.

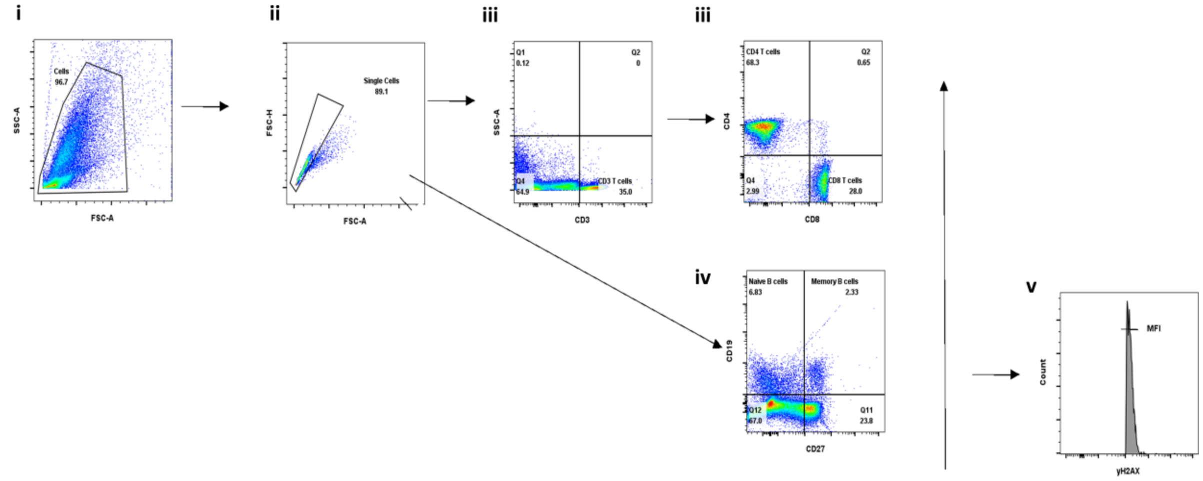


**Supplementary Figure S3: γ-H2AX assay gating strategy.** Left-to-right: (i) FSCA × SSCA to select the total cell population and exclude debris, (ii) FSCH × FSCA doublet discrimination gate, (iii) lineage cocktail gate separating $CD3^+$ cells into $CD4^+$ T cells and $CD8^+$ T cells. (iv) Single cells were re-gated again for naïve B cells ($CD19^+$ $CD27^-$), and memory B cells ($CD19^+$ $CD27^+$). (v) Histogram overlay of γ-H2AX- Alexa 488 median fluorescence intensity (MFI) for each lineage at the indicated concentration and time.

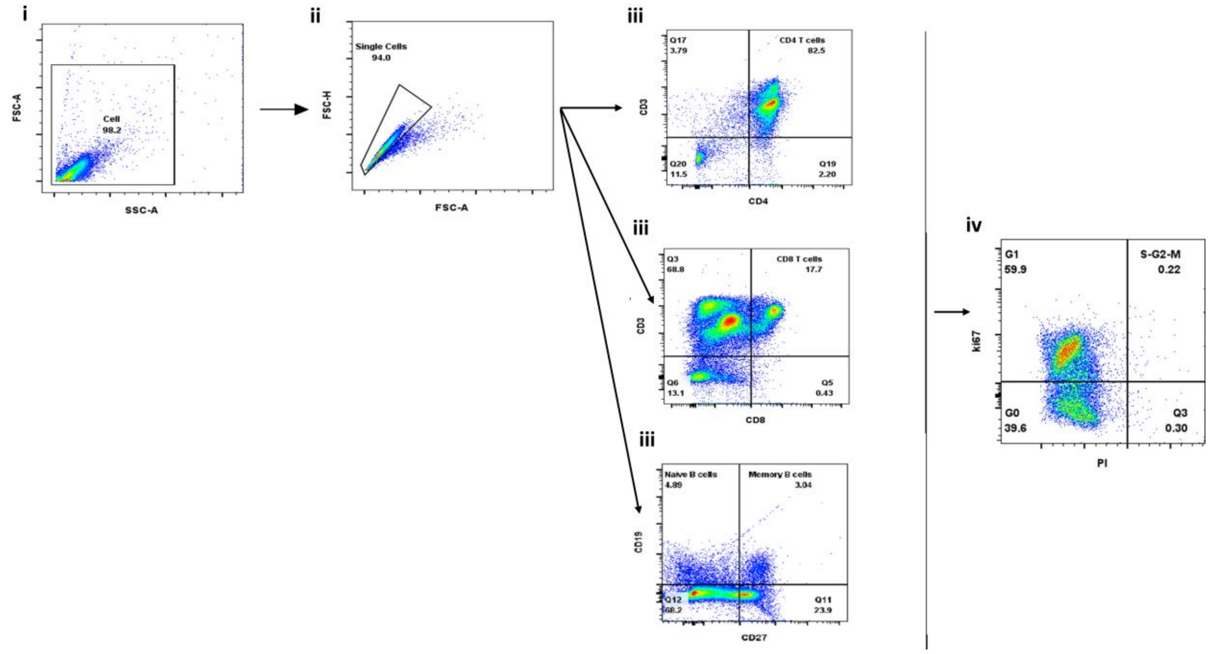


**Supplementary Figure S4: Ki-67-FITC/propidium iodide (PI) cell cycle assay gating strategy.** Left-to-right: (i) FSCA × SSCA to select the total cell population and exclude debris, (ii) FSCH × FSCA doublet discrimination gate, (iii) lineage cocktail gate separating $CD4^+$ T cells ($CD3^+CD4^+$), $CD8^+$ T cells ($CD3^+CD8^+$), naïve B cells ($CD19^+$ $CD27^-$), and memory B cells ($CD19^+$ $CD27^+$), (iv) bivariate plot of Ki-67-FITC versus PI fluorescence with quadrant gates defining $G_0$ ($Ki67^-$ / 2N DNA), $G_1$ ($Ki67^+$ / 2N), and S-$G_2$-M (Ki-67 $^+$/ >2N) fractions. Gates were applied identically across all treatment groups and time points. Data were acquired on a CytoFLEX LX (Beckman Coulter) and analysed in FlowJo v10.9.

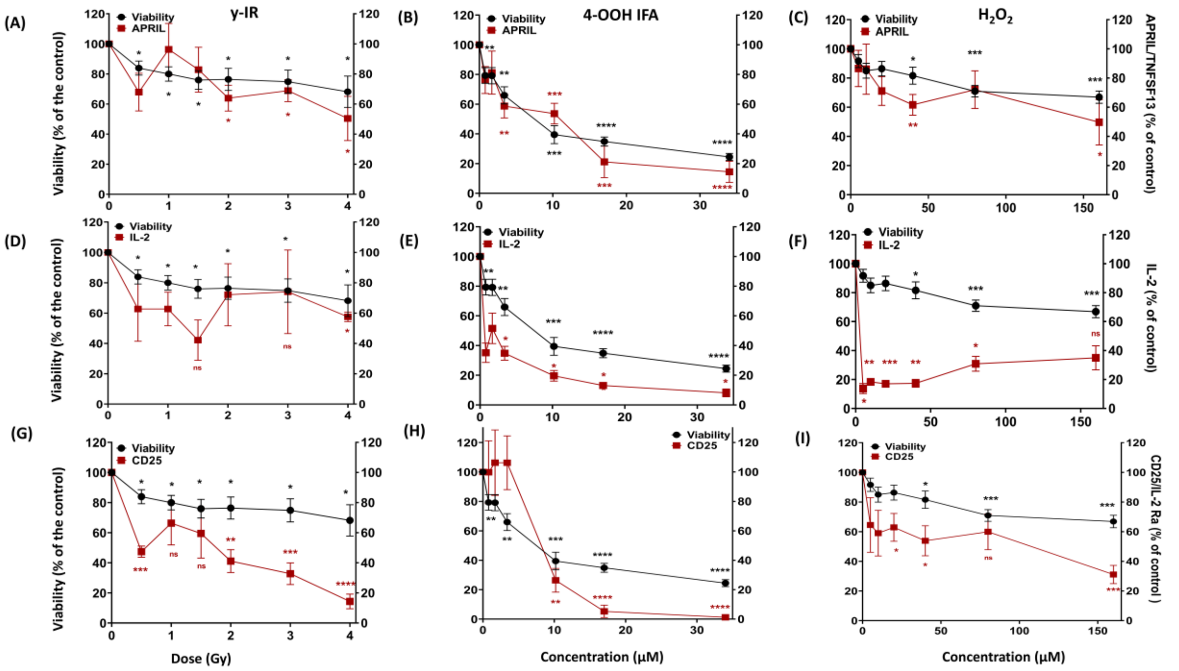


**Supplementary Figure S5: Extended cytokine/chemokine panel concentration response curves**. Day 8 (7 days post-treatment) supernatant dose/concentrations responses for APRIL (A-C), IL-2 (D-F), and soluble sCD25/IL-2 Ra (G-I) in PBMC-RA-FLS co-cultures (red), plotted against the matched day 8 viability (black) of the PBMC-RA-FLS co-cultures from Fig. 1 for γ-IR (A, D, G), 4-OOH IFA (B, E, H), and $H_2O_2$ (C, F, I). Values are expressed as % of CpG-stimulated untreated control. The viability curve (black line, secondary y-axis) is identical across all panels and represents day 8 viability from the same wells used for the corresponding supernatant measurements. Spearman correlations with viability are summarized in Supplementary Table S4. Statistics: repeated-measures one-way ANOVA with Dunnett's post hoc vs untreated control. *$p \leq 0.05$, **$p \leq 0.01$, ***$p \leq 0.001$, ****$p \leq 0.0001$; ns, not significant. PBMC-RA-FLS: *N = 3, n = 2*

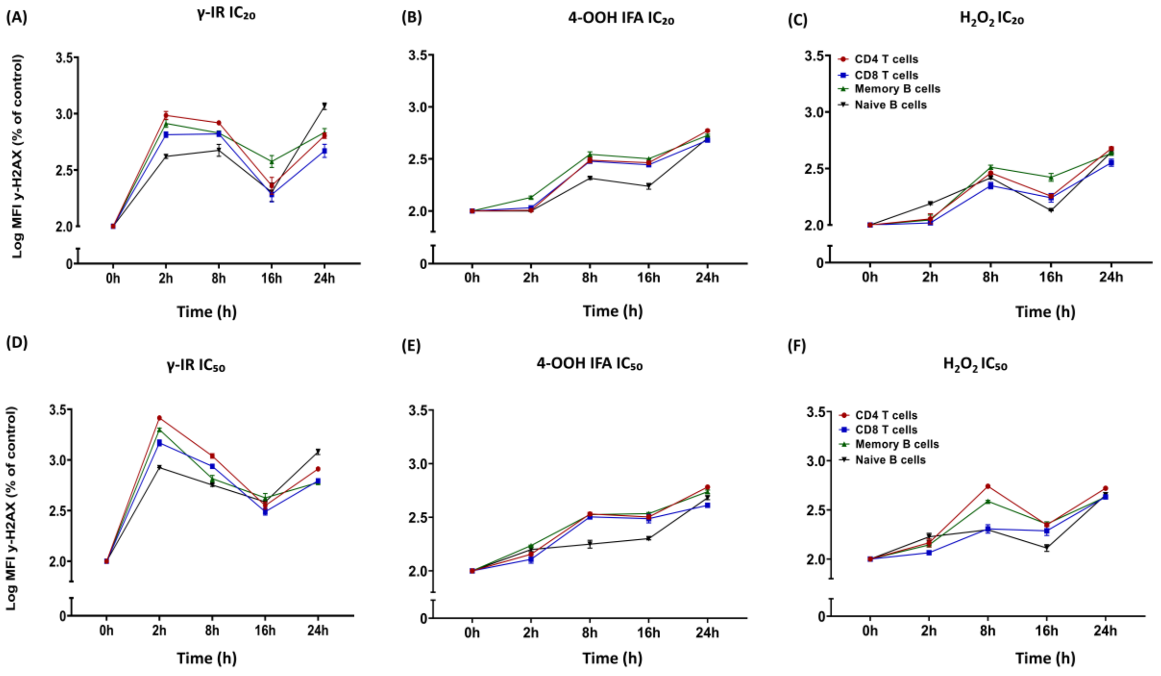


**Supplementary Figure S6: Log-scaled γ-H2AX kinetics for each lineage and stressor.** IC$_{20}$ (A-C) and IC$_{50}$ (D-F) time courses (2, 8, 16, 24 h) of log (γ-H2AX median fluorescence intensity (MFI) in gated CD4$^+$ T (red), CD8$^+$ T (blue), memory B (CD19$^+$CD27$^+$) (green), and naïve B (CD19$^+$CD27$^-$) (black) cells within PBMC-RA-FLS co-culture after γ-IR (A, D), 4-OOH IFA (B, E), or $H_2O_2$ (C, F). MFI is normalized to the time-matched untreated control. These panels complement central Figure 4 by displaying the same data on a log$_{10}$ axis to emphasise peak spreading and convergence. IC$_{20}$/IC$_{50}$ doses/concentrations are as defined in Figure 1 (Supplementary Table S2). Gating strategy: Supplementary Fig. S3; Statistics: repeated-measures one-way ANOVA with Dunnett's post hoc vs time-matched control at each timepoint. *$p \leq 0.05$, **$p \leq 0.01$, ***$p \leq 0.001$, ****$p \leq 0.0001$; ns, not significant. Adjusted p-values for comparisons to the 0-hour baseline within each lineage are reported in Supplementary Table S5. PBMC-RA-FLS: *N = 4, n = 1*

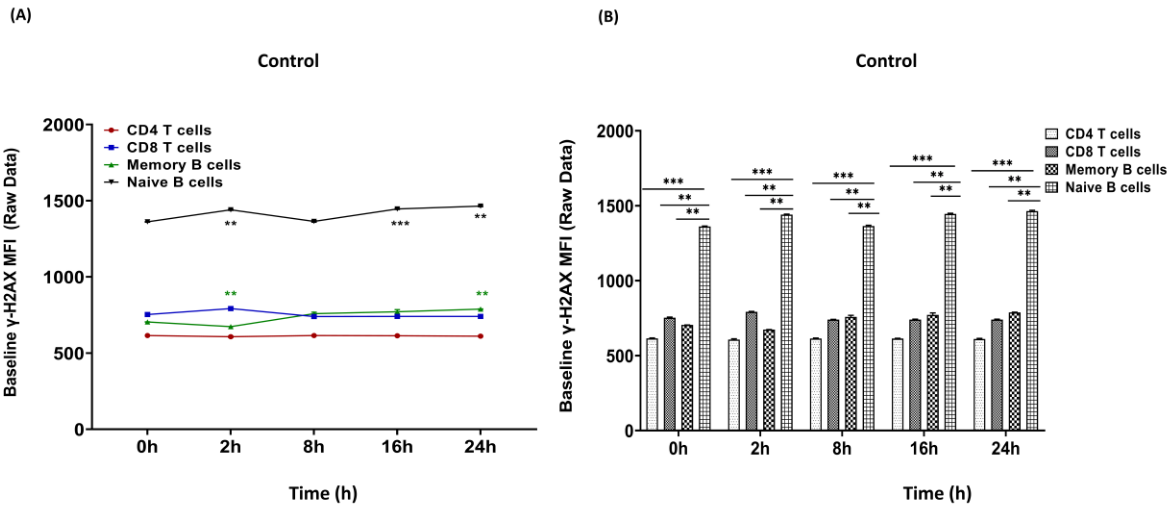


**Supplementary Figure S7: Absolute γ-H2AX MFI values for control untreated samples**. (A) time courses (2, 8, 16, 24 h) of absolute γ-H2AX median fluorescence intensity (MFI) in gated $CD4^{+}$ T (red), $CD8^{+}$ T (blue), memory B ($CD19^{+}CD27^{+}$) (green), and naïve B ($CD19^{+}CD27^{-}$) (black) control untreated cells of PBMC-RA-FLS co-culture. (B) Bar graphs show absolute median fluorescence intensity (MFI) of phosphorylated histone H2AX (Ser139) in $CD4^{+}$ T cells, $CD8^{+}$ T cells, memory ($CD19^{+}$ $CD27^{+}$) B cells, and naïve ($CD19^{+}$ $CD27^{-}$) B cells 0-24 hours after treatment. $IC_{20}/IC_{50}$ doses/concentrations are as defined in Figure 1 (Supplementary Table S2). Data are mean ± SEM. Statistics: repeated-measures one-way ANOVA within each lineage followed by Dunnett's post hoc test versus control .* $p \leq 0.05$, ** $p \leq 0.01$, *** $p \leq 0.001$, **** $p \leq 0.0001$; ns, not significant. PBMC-RA-FLS: $N = 4, n = 1$ .

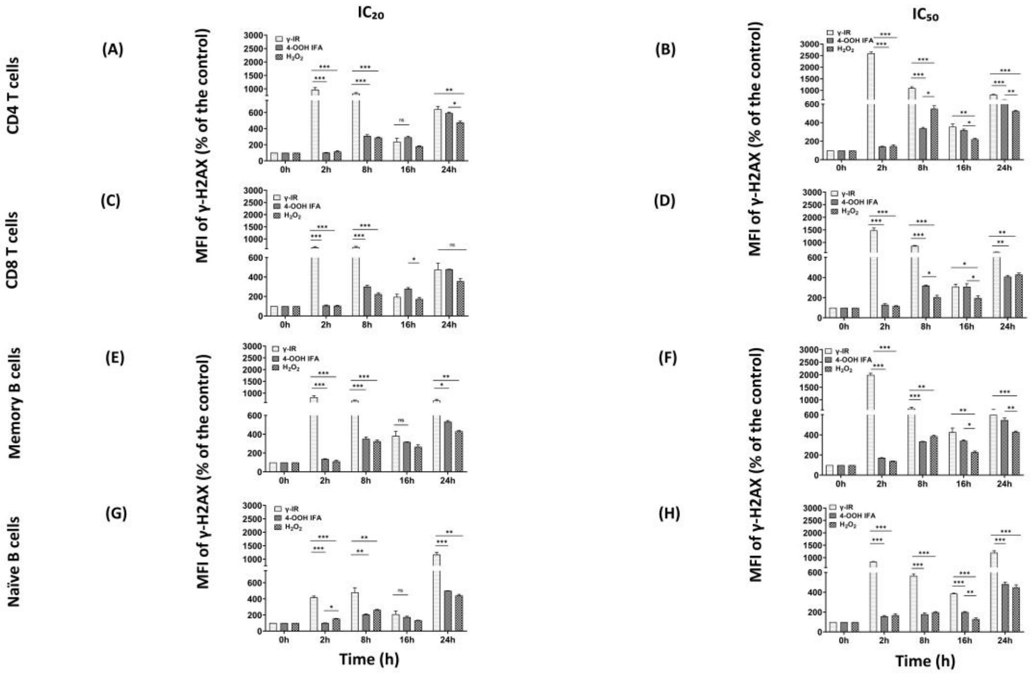


**Supplementary Figure S8: Comparison of γ-H2AX DNA-damage signal for each treatment within immune subsets (0-24 hours).** Bar graphs show median fluorescence intensity (MFI) of phosphorylated histone H2AX (Ser139) in CD4$^+$ T cells, CD8$^+$ T cells, memory (CD19$^+$ CD27$^+$) B cells, and naïve (CD19$^+$ CD27$^-$) B cells within PBMC-RA-FLS co-culture 0-24 hours after treatment, normalised to the time-matched untreated control. For each lineage, bars represent the indicated treatment(s) at the viability-matched concentrations ([IC$_{20}$] and/or [IC$_{50}$]) defined in Fig. 1, with values normalized to the time-matched untreated control. IC$_{20}$/IC$_{50}$ doses/concentrations are as described in Figure 1 (Supplementary Table S2). Data are mean ± SEM. Statistics: repeated-measures one-way ANOVA within each lineage followed by Dunnett's post hoc test versus control. * $p \leq 0.05$, ** $p \leq 0.01$, *** $p \leq 0.001$, **** $p \leq 0.0001$; ns, not significant. PBMC-RA-FLS: $N = 4, n = 1$

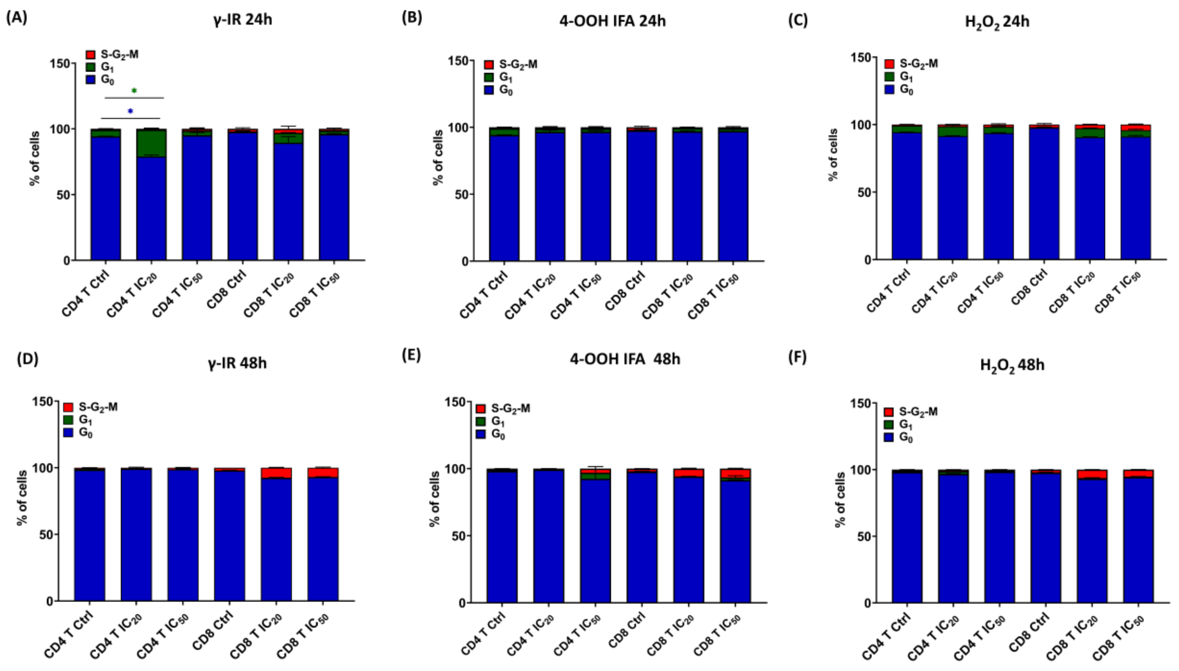


**Supplementary Figure S9: CD4⁺ and CD8⁺ T cell Ki-67-FITC/PI profiles.** Analogous to Figure 5, the transient and fully reversible checkpoints in T cell subsets are shown. Ki-67-FITC/propidium iodide (PI) profiles at 24 hours after the single [$IC_{20}$] or [$IC_{50}$] pulse (A-C) and 48 hours (i.e., 24 hours after CpG stimulation, D-F). For each time point, the three stressors are shown side by side: γ-IR (A, D), 4-OOH IFA (B, E), and $H_2O_2$ (C-F). Within each stressor, the left-hand bars represent $CD4^+$ T cells, and the right-hand bars represent $CD8^+$ T cells gated within PBMC-RA-FLS co-culture. Stacked columns display mean ± SEM percentages of cells in $G_0$ (blue), $G_1$ (green), and S-$G_2$-M (red) phases. $IC_{20}$/$IC_{50}$ doses/concentrations are as defined in Figure 1 (Supplementary Table S2). Statistics: repeated-measures one-way ANOVA with Dunnett's post hoc vs untreated control. *$p \leq 0.05$, **$p \leq 0.01$, ***$p \leq 0.001$, ****$p \leq 0.0001$; ns, not significant. PBMC-RA-FLS: *N = 4, n = 1*

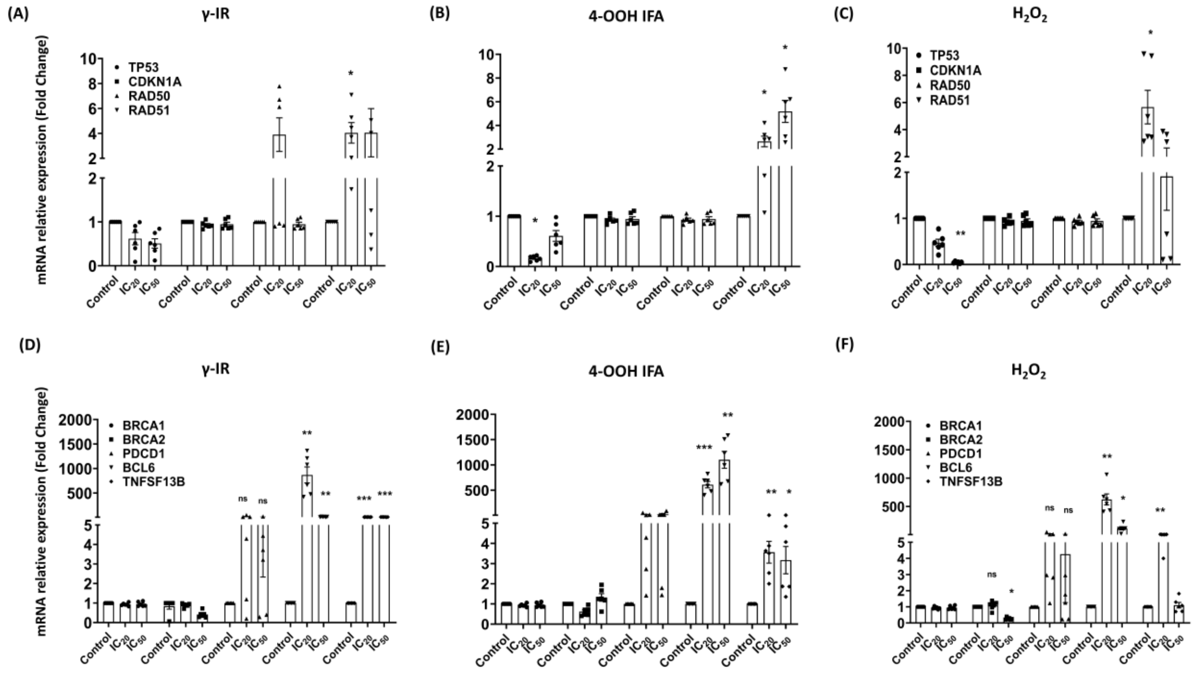


**Supplementary Figure S10: DNA-damage/checkpoint regulators and B cell survival factors at 24 h by RT-qPCR.** Bar charts show mRNA fold change (ΔΔCt) relative to time-matched untreated control of whole PBMC-RA-FLS co-culture, measured 24 hours after a single $IC_{20}$ or $IC_{50}$ dose/concentration of γ-IR (A, D), 4-OOH IFA (B, E), or $H_2O_2$ (C, F) (mean ± SEM). Upper row (A-C): RAD50, RAD51, TP53, CDKN1A (p21). Lower row (D-F): BRCA1, BRCA2, PDCD1, BCL6, and TNFSF13B. It complements the regulator panel in Figure 6. $IC_{20}$/$IC_{50}$ doses/concentrations are as defined in Figure 1 (Supplementary Table S2). Statistics: repeated-measures one-way ANOVA with Dunnett's post hoc vs control. *p ≤ 0.05, **p ≤ 0.01, ***p ≤ 0.001, ****p ≤ 0.0001; ns, not significant. PBMC/RA-FLS: *N = 3, n = 2.*

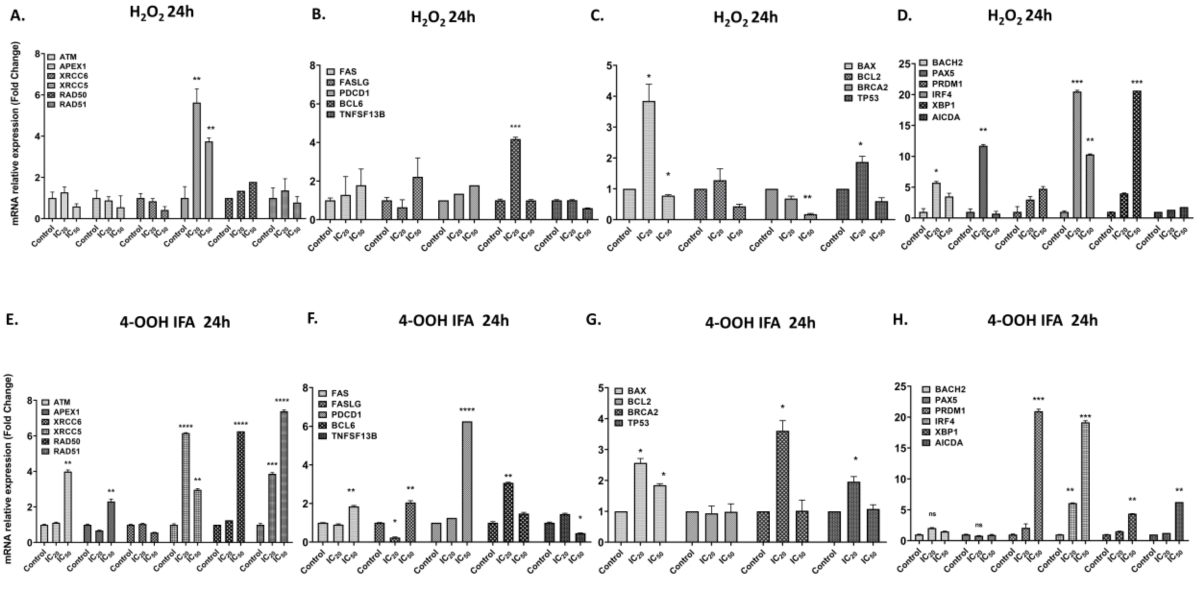


**Supplementary Figure S11: RA-FLS monocultures: 24 h RT-qPCR profile across gene modules after oxidative or alkylating stress**. RA-FLS monocultures ("FLS only", no PBMCs) were exposed once to $H_2O_2$ (A-D) or 4-OOH ifosfamide (4-OOH IFA) (E-H) at the indicated concentrations (matching the nominal [$IC_{20}$]/[$IC_{50}$] levels defined in Fig. 1 for the co-culture). Bars show mean ± SEM, N = 3 independent RA-FLS donors. Panel map (gene grouping by pathway): (A, E): DNA damage sensors/repair: ATM, APEX1, XRCC6 (Ku70), XRCC5 (Ku80), RAD50, RAD51. (B, F) Late cell differentiation effector readout/apoptosis (survival and heavy-chain transcripts): BCL6, TNFSF13B, FAS, FASL, PDCD1, IGHM, IGHG1, IGHA1 (Ig transcripts were near or below detection). (C, G) Cell cycle checkpoint/apoptosis/co-inhibitory: TP53, CDKN1A (p21), BAX, BCL2, BRCA1, BRCA2. (D, H) B cell differentiation cassette (reported here in FLS as off-target/trans-effects controls): BACH2, PAX5, IRF4, PRDM1, XBP1, AICDA. Statistics were performed within each panel using repeated-measures one-way ANOVA with Dunnett's post hoc test versus the untreated control: $*p \leq 0.05$, $**p \leq 0.01$, $***p \leq 0.001$, $****p \leq 0.0001$; ns, not significant. RA-FLS: *N = 3, n=1*

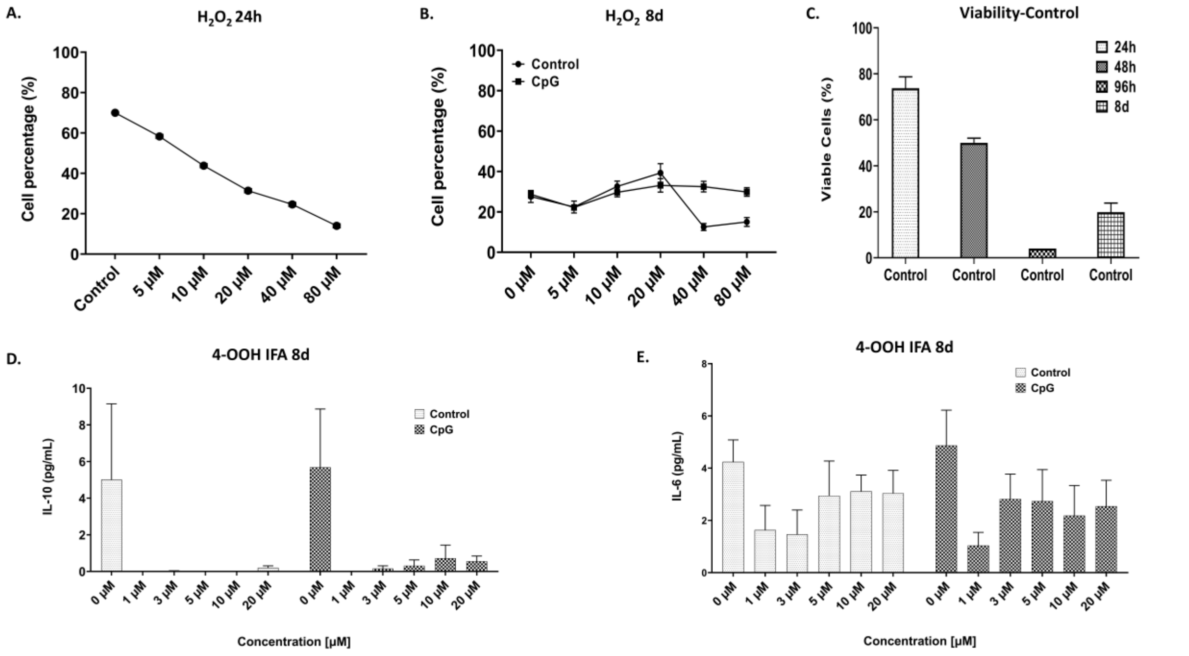


**Supplementary Figure S12. Viability and cytokine production of PBMCs only treated with genotoxic stressors.** (A) Peripheral blood mononuclear cells (PBMCs) cultured alone were treated with increasing concentrations of hydrogen peroxide ($H_2O_2$) for 24 h, showing a concentration-dependent decrease in viable cells. (B) PBMCs cultured alone for 8 days with or without CpG were exposed to the indicated $H_2O_2$ concentrations, and cell viability was assessed at day 8. (C) Time-course of PBMC viability under control conditions (no genotoxic treatment) at 24 h, 48 h, 96 h, and 8 days. (D-E) At day 8, PBMCs cultured alone and pre-treated with 4-OOH IFA were analyzed for cytokine production, showing IL-10 (D) and IL-6 (E) levels.

**Supplementary Table S1:** All used compounds and Antibodies for the study.

| Antibody/Compound/Kit | Fluorochrome | Species | Company | Catalog Nr. | Final Concentration |
|---|---|---|---|---|---|
| **CD19** | efluor 405 | Human | Invitrogen | # 48-0198-42 | 1:20 |
| **CD27** | VioBright R720 | Human | Miltenyi Biotec | 130-128-404 | 1:50 |
| **CD4** | PerCP/Vio700 | Human | Miltenyi Biotec | 130-113-228 | 1:50 |
| **CD8** | VioGreen | Human | Miltenyi Biotec | 130-110-684 | 1:50 |
| **CD3** | PE/Vio770 | Human | Miltenyi Biotec | 130-113-140 | 1:50 |
| **γ-H2AX** | Alexa Fluor 488 | Human | BD Biosciences | 560445 | 1:20 |
| **Ki-67** | FITC | Human | Miltenyi Biotec | 130-117-691 | 1:50 |
| **PI** | - | Human | Miltenyi Biotec | 130-093-233 | 1:100 |
| **Annexin V/PI kit** | FITC | Human | Miltenyi Biotec | 130-092-052 | |
| **Fcr blocking reagent** | | Human | Miltenyi Biotec | 130-059-901 | 1:5 |
| **γ-IR: Gammacell 100 Elite radiation machine** | | | Nordion International | | |
| **4-OOH IFA** | | | Niomech IIT GmbH | D-18851 | |

| | | | | | |
|---|---|---|---|---|---|
| **$H_2O_2$** | | | Sigma-Aldrich | 7722-84-1 | |
| **CpG ODN 2006** | | Human | Invivogen | tlrl-2006-1 | 5 µg/mL |
| **Human IL-10 ELISA set** | | Human | BD Biosciences | 555157 | |
| **Human IFN-γ ELISA set** | | Human | BD Biosciences | 555142 | |
| **Human APRIL/TNFSF13 DuoSet ELISA** | | Human | R&D | DY884B | |
| **Human IL-2 DuoSet ELISA** | | Human | R&D | DY202-05 | |
| **Human IL-2 Ra/CD25 DuoSet ELISA** | | Human | R&D | DY223 | |
| **Human IgM ELISA Antibody Pair Kit** | | Human | StemCell | #01995A | |
| **Human IgG ELISA Antibody Pair Kit** | | Human | StemCell | #01994A | |
| **Human IgA ELISA Antibody Pair Kit** | | Human | StemCell | #01992A | |
| **Human IgE ELISA Antibody Pair Kit** | | Human | StemCell | #01993A | |
| **Primers** | | Human | Eurofins Genomics | | |

**Supplementary Table S2 :** Relative cytotoxic $IC_{50}$ values (24 h and 8 d) unless stated otherwise, such as γ-irradiation $IC_{20}$, 4-hydroperoxyifosfamide $IC_{20}$, or hydrogen peroxide $IC_{20}$, which refers to relative $IC_{20}$ values and not relative $IC_{50}$ derived from the four-parameter fits in main Figure 1.

| **Time** | **Treatment** | **Relative $IC_{50}$ (µM/Gy)** |
|---|---|---|
| **24h** | γ-Irradiation | 0.4 Gy |
| **8d** | γ-Irradiation | 0.9 Gy |
| **24h** | 4-hydroperoxyifosfamide | 14 µM |
| **8d** | 4-hydroperoxyifosfamide | 7 µM |
| **24h** | Hydrogen Peroxide | 23 µM |
| **8d** | Hydrogen Peroxide | 60 µM |
| **24h** | 4-hydroperoxyifosfamide RA -FLS | 7.7 µM |
| **24h** | Hydrogen Peroxide RA- FLS | cannot calculate |
| **24h** | γ-Irradiation- $IC_{20}$ | 0.1 Gy |
| **24h** | 4-hydroperoxyifosfamide- $IC_{20}$ | 3.5 µM |
| **24h** | Hydrogen Peroxide - $IC_{20}$ | 6 µM |

**Supplementary Table S3.** Primers used for RT-qPCR. All primer sequences are listed from 5′ to 3′. Primer sets comprise in-house designed primers, commonly used primer pairs adopted from established RT-qPCR literature, and primers previously reported in related studies (e.g. Ouzin et al. 2024) (1). All primers were re-validated in silico for specificity and experimentally confirmed in this study to yield single, specific amplicons under the conditions used.

| Gene | Forward Primer (5'-3') | Reverse Primer (5'-3') |
|---|---|---|
| ***ATM*** | *TTACGGGTGTTGAAGGTGTCT* | *GGATTCATGGTCCAGTCAAAG* |
| ***APEX1*** | *CTGCCTGGACTCTCTCATCAATAC* | *CCTCATCGCCTATGCCGTAAG* |
| ***XRCC6*** | *AAAAGACTGGGCTCCTTGGT* | *TGTGGGTCTTCAGCTCCTCT* |
| ***XRCC5*** | *CGACAGGTGTTTGCTGAGAA* | *GAATCACATCCATGCTCACG* |
| ***RAD50*** | *CTTGGATATGCGAGGACGAT* | *CGCATTGAAGGTCGAAGACC* |
| ***RAD51*** | *GCCACCGCCCTTTACAGAACA* | *TGGGATCAGCAGCAAACATCG* |
| ***BRCA1*** | *CCACAGATCAACCTGGAATGG* | *GTAGAGTGCTACACTGCTCA* |
| ***BRCA2*** | *TTCTGAGGTGGACCTAATAGG* | *TGATTTGGATTCTGGTCGCC* |
| ***FAS*** | *GACCCTCCTACCTCTGGTTCTT* | *TGTGCAGTCCCTAGCTTTCC* |
| ***FASLG*** | *TCTACCAGCCAGATGCACAC* | *CTTGAGTTGGACTTGCCTGT* |
| ***BAX*** | *GCCCTTTTGCTTCAGGGTTT* | *TCCAATGTCCAGCCTTTG* |
| ***BCL2*** | *GAAGCATACCCGTTTAGC* | *CGAGAACTGGGAGAAGAA* |
| ***TP53*** | *TTCCGAGAGCTGAATGAGGC* | *AATGTCAGTCTGAGTCAGGCC* |
| ***CDKN1A*** | *TACATCTTCTGCCTTAGT* | *TCTTAGGAACCTCTCATT* |
| ***PDCD1*** | *CGTGACTTCCACATGAGCGT* | *CTGGCTCCTATTGTCCCTCGT* |
| ***BCL6*** | *AGGCCGGACACCAGGTTTTG* | *GCTCTAAACTGCTCACGGCT* |
| ***TNFSF13B*** | *GTGAAACACCAACTATACAAAAAGG* | *GTTTTGCAATGCCAGCTGAA* |
| ***BACH2*** | *CGTTCGTTCACATAGCTCCCA* | *TCAGTGAGTGTCCACCTTGTTC* |
| ***XBP1*** | *TCTGGAGCTATGGTGGTGGT* | *GTTTCCTCCTCAGCGCCTT* |
| ***IRF4*** | *CCCAGCTTGTGAAAATGGTTG* | *ACCTTATGCTTGGCTCTGTGG* |
| ***PAX5*** | *ATTTCACGGTGCCTTCGGAC* | *TCACTCCTCCATGTCCTGTC* |
| ***PRDM1*** | *CTCCAGTGTTGCGGAGAGG* | *GGGGCAGCCAAGGTCG* |
| ***AICDA*** | *CGCATCCTTTTGCCCCTGTA* | *GCCAGACCTGTGTTCCTTCT* |
| ***IGHM*** | *GCTGAGGCAAAGGAGTCTG* | *TGGTCTGCTTCAGTGGCG* |

| | | |
|---|---|---|
| ***IGHA1*** | *ACCATGCAGGAGAAGGTGTC* | *TCACTTGCACTGCTGCCTAC* |
| ***IGHG1*** | *GCAGCCGGAGAACAACTACA* | *TGGTTGTGCAGAGCCTCGAT* |
| ***IL10*** | *GGAAGCCAGGATCACCAACA* | *CCTTCCATGCTTTGGGGTTG* |
| ***TLR9*** | *GCCAGACCCTCTGGAGAA* | *GGCACAGTCATGATGTTGTTGTA* |
| ***r18S*** | *TAACCCGTTGAACCCCATT* | *CCATCCAATCGGTAGTAGCG* |

**Supplementary Table S4:** Spearman rank correlations (ρ [p]) between day-8 viability and functional read-outs (IL-10, IFN-γ, APRIL, sCD25, IL-2, IgG, IgA, IgM).

| **Read-out** | **γ-IR-Viability-day 8 ρ (*p*)** | **4-OOH IFA- Viability-day 8 ρ (*p*)** | **$H_2O_2$- Viability-day 8 ρ (*p*)** |
|---|---|---|---|
| **IL-10** | **0.964 (0.003)** | **0.895 (0.007)** | **0.923 (0.003)** |
| **IFN-γ** | 0.750 (0.066) | **0.893 (0.009)** | **0.925 (0.003)** |
| **IgG** | **0.972 (0.002)** | **0. 953 (0.001)** | 0.257 (0.623) |
| **IgA** | 0.428 (0.354) | **0.972 (0.0002)** | 0.526 (0.064) |
| **IgM** | **0.857 (0.013)** | **0.898 (0.006)** | 0.513 (0.238) |
| **APRIL** | 0.735 (0.059) | **0.958 (0.007)** | **0.856 (0.014)** |
| **sCD25** | **0.821 (0.0341)** | **0.9162 (0.0037)** | **0.893 (0.012)** |
| **IL-2** | 0.742 (0.056) | **0.899 (0.006)** | –0.214 (0.645) |

**Supplementary Table S5:** Adjusted *p*-values for γ-H2AX MFI: treated versus time-matched control (One way ANOVA + Dunnett) across four lineages (CD4 T, CD8 T, memory B, naïve B cells), four time points (2 h, 8 h, 16 h, 24 h) and two doses/concentrations ($IC_{20}$, $IC_{50}$) for each stressor. Significant values are bold in the spreadsheet.

| **Cell Type** | | **CD4 T cell** | **CD8 T cell** | **Memory B cell** | **Naïve B cell** |
|---|---|---|---|---|---|
| **Treatment** | **Timepoint** | **Adjusted p.value** | | | |
| | | | | | |
| **γ-IR $IC_{20}$** | **2h** | **0.0172** | **0.0090** | **0.0172** | **0.0062** |
| | **8h** | **0.0040** | **0.0081** | **0.0054** | **0.0501** |
| | **16h** | 0.1882 | 0.1555 | 0.0629 | 0.2479 |
| | **24h** | **0.0092** | 0.0643 | **0.0174** | **0.0125** |
| **γ-IR $IC_{50}$** | **2h** | **0.0012** | **0.0109** | **0.0028** | **<0.0001** |
| | **8h** | **0.0072** | **0.0010** | **0.0177** | **0.0034** |
| | **16h** | **0.0286** | **0.0267** | **0.0348** | **0.0010** |
| | **24h** | **0.0012** | **<0.0001** | **0.0034** | **0.0093** |
| **4-OOH IFA $IC_{20}$** | **2h** | 0.0851 | 0.4705 | **0.0261** | **>0.0999** |
| | **8h** | **0.0147** | **0.0108** | **0.0126** | **0.0070** |
| | **16h** | **0.0078** | **0.0114** | **0.0001** | **0.0499** |
| | **24h** | **0.0016** | **<0.0001** | **0.0024** | **<0.0001** |
| **4-OOH IFA $IC_{50}$** | **2h** | **0.0096** | 0.2448 | **0.0012** | **0.0326** |
| | **8h** | **0.0049** | **0.0019** | **<0.0001** | 0.0703 |
| | **16h** | **0.0074** | **0.0396** | **0.0028** | **0.0063** |
| | **24h** | **0.0002** | **0.0034** | **0.0044** | **0.0058** |
| **$H_2O_2$ $IC_{20}$** | **2h** | 0.6530 | 0.7292 | 0.7209 | **0.0047** |
| | **8h** | **0.0033** | **0.0306** | **0.0082** | **0.0042** |
| | **16h** | **0.0103** | 0.0835 | **0.0376** | **0.0021** |
| | **24h** | **0.0040** | **0.0212** | **0.0013** | **0.0034** |

| | | | | | |
|---|---|---|---|---|---|
| **$H_2O_2$ $IC_{50}$** | **2h** | 0.1629 | 0.0999 | **0.0015** | 0.0846 |
| | **8h** | **0.0102** | 0.0683 | **0.0018** | **0.0084** |
| | **16h** | **0.0103** | 0.1106 | **0.0177** | 0.2111 |
| | **24h** | **0.0007** | **0.0051** | **0.0016** | **0.0118** |

**References:**

1. Ouzin M, Wesselborg S, Fritz G, Kogler G. Evaluation of Genotoxic Effects of N-Methyl-N-Nitroso-Urea and Etoposide on the Differentiation Potential of MSCs from Umbilical Cord Blood and Bone Marrow. Cells. 2024;13(24).
